# Landscape heterogeneity affects developmental and dispersal‐related traits of a butterfly in agricultural landscapes

**DOI:** 10.1002/eap.70209

**Published:** 2026-03-11

**Authors:** Franziska Deppe, Emily Breuer, Inka Hofmann, Nicla Koch, Lara Näckel, Josua Nowak, Philip Carlo Plänker, Anna‐Lena Schmitz, Lisa Schroeder, Anna Spitzlei, Paula Vetter, Lukas Wassong, Stefanie Weich, Michael Weingart, Luisa Wittkamp, Mine Yilmazer, Klaus Fischer

**Affiliations:** ^1^ Institute for Integrated Natural Sciences, Zoology University of Koblenz Koblenz Germany; ^2^ Present address: Institute for Landscape Ecology and Resources Management (ILR), Research Centre for BioSystems, Land Use and Nutrition (iFZ), Justus Liebig University Giessen Giessen Germany

**Keywords:** agricultural intensification, *Coenonympha pamphilus*, food stress, habitat fragmentation, insect declines, landscape configuration, life history traits, population responses

## Abstract

The loss and fragmentation of natural habitats due to the intensification of agricultural land use have detrimental impacts on the biodiversity of arthropods. The reduction of natural habitats results in a decreased availability of essential resources, which may select for rapid development and phenotypes enhancing dispersal ability. We here compared replicated populations of the butterfly *Coenonympha pamphilus* in field‐caught females and their laboratory‐reared offspring across two landscape types: highly fragmented and intensified “modern” and less fragmented “traditional” agricultural landscapes. We also examined the effects of food stress and landscape parameters representing compositional and configurational landscape heterogeneity on intraspecific trait variation at different spatial scales. The differences between the two landscape types in butterfly traits were nonsignificant throughout, but both field‐caught females and their offspring exhibited various responses to the measured landscape parameters. In particular, landscapes with (1) high heterogeneity of habitat patches (i.e., relatively smaller grassland patches with high boundary length), (2) higher proportion of non‐crop habitats (i.e., grassland, forests, and woodland), and (3) lower proportion of crop fields seemed to select for phenotypes enhancing dispersal ability. Flight propensity of male offspring was increased under food stress, indicating plastic responses to resource scarcity. In conclusion, our findings suggest that the compositional and configurational landscape heterogeneity, namely parameters indicative of agricultural intensification, select for enhanced dispersal in *C. pamphilus*. As higher investment in dispersal often comes at a cost to reproduction, such trait shifts may reduce population viability, which may have important implications for insect declines in agricultural landscapes.

## INTRODUCTION

Habitat fragmentation, originally defined as the process of dividing large, continuous habitats into smaller, isolated patches (Wilcove et al., 1986), represents a significant driver of community composition and species survival (Benton et al., 2003; Fahrig, 2017; Saura et al., 2014). With the conversion of natural landscapes into agricultural or urban areas, remaining habitats become increasingly fragmented, which may result in isolated relict populations and possibly disrupted ecological processes (Didham, 2010; Prugh et al., 2008). Intensive agriculture often results in monocultures and a widespread use of pesticides and fertilizers, reducing habitat and resource availability, thereby exacerbating the effects of habitat fragmentation (Emmerson et al., 2016; Ghazali et al., 2016; Tscharntke et al., 2005; Zou et al., 2017). As a consequence, the decline of arthropods, including butterflies, in agricultural landscapes has been extensively documented (Habel et al., 2019; Outhwaite et al., 2022; Seibold et al., 2019; Wagner et al., 2021).

In intensively managed agricultural landscapes, butterfly communities are mainly comprised of generalist species with strong dispersal abilities, whereas more specialized and less mobile species tend to decline or even disappear (Börschig et al., 2013; Guariento et al., 2023; Habel et al., 2019). Such interspecific differences reflect that, across species, dispersal‐related morphological and behavioral traits (e.g., wing size, flight musculature, or exploratory behavior) determine which species can persist in fragmented landscapes. For the survival of species in fragmented landscapes, dispersal ability is thus crucial as it determines the capacity of individuals to move between habitat patches, locate resources, and maintain genetic diversity (Fahrig et al., 2011; Priyadarshana et al., 2024). Consequently, morphological traits enhancing dispersal ability should be favored in fragmented landscapes (Gámez‐Virués et al., 2015; Neff et al., 2019).

At the intraspecific level, that is, among populations of the same species, previous studies focusing on dispersal in flying insects in relation to habitat fragmentation found different responses to landscape heterogeneity (Clobert, 2012; Cote et al., 2017). However, flight capacity and habitat finding ability of butterflies were demonstrated to increase with increasing fragmentation or isolation of habitats (Lebeau et al., 2016a; Merckx & Van Dyck, 2007). Similarly, the loss of habitat area, which is often linked to habitat fragmentation, has been shown to increase wing size, thorax or body mass in flying insects across populations (Lion et al., 2022; Taylor & Merriam, 1995; Thomas et al., 1998). Hence, intraspecific variability in flight capacity may be influenced by inherited morphological traits such as the thorax–abdomen ratio or wing size (Betts & Wootton, 1988; Ducatez, Legrand, et al., 2012b; Jahant‐Miller et al., 2022). Flight propensity, though, that is, the willingness to fly, was found to either decrease (Merckx et al., 2003; Schtickzelle et al., 2006) or increase (Lebeau et al., 2016a; Reim, Baguette, et al., 2018a) with increasing habitat fragmentation, potentially depending on the test performed. Flight propensity might also be shaped by inter‐individual differences in exploratory behavior, being influenced by external factors such as resource distribution and thus food availability (Baguette et al., 2011; Reim, Baguette, et al., 2018a; Saastamoinen et al., 2010). In addition to dispersal‐related traits, other life history traits may also be affected by habitat fragmentation in butterflies. In particular, populations inhabiting fragmented landscapes may experience selection favoring shorter life spans or faster development (Duplouy et al., 2013). Such adaptations may affect survival and reproductive success (Violle et al., 2007), thereby shaping the overall evolutionary trajectory of populations including dispersal strategies.

In a previous study, we investigated the effect of the landscape composition and configuration on trait variation in field‐caught males of *Coenonympha pamphilus*. We showed that landscapes with a high proportion of crop fields were associated with phenotypes that enhance dispersal ability (Deppe et al., 2024). However, trait variation in response to changing landscapes may result from genetic adaptation or phenotypic plasticity (Chuang & Peterson, 2016). Genetic adaptation may involve heritable changes in traits conferring a survival advantage in fragmented habitats (Hill et al., 1999), while phenotypic plasticity refers to the ability of an organism to alter its morphology or behavior within a given genotype, induced by environmental variation (Nijhout, 1999). Distinguishing between the two, adaptation and plasticity, may be crucial for the conservation and management of insect populations. To disentangle environmental effects (i.e., phenotypic plasticity) from genetic adaptation, we here investigate landscape effects on butterfly traits in field‐caught females and their offspring in the butterfly *Coenonympha pamphilus*, using a common garden experiment for the latter. We included the field‐caught females to test whether patterns are consistent with those found in their offspring, which would indicate a heritable component. Divergent patterns, in contrast, would indicate environmental impacts and thus plasticity. To this end, we compared replicated populations originating from “modern” and “traditional” agricultural landscapes, differing in overall compositional and configurational landscape heterogeneity. Furthermore, we included different adult feeding treatments for the offspring to elucidate the role of adult income for storage reserves, flight performance, and reproductive investment, as well as the extent to which fitness‐related traits are susceptible to reduced nectar availability.

We address our following predictions at the level of landscape type (i.e., modern vs. traditional) and in relation to landscape composition and configuration, that is, landscape features (e.g., crop field size) indicative of agricultural intensification and habitat fragmentation.

We predict that (H1) developmental traits in offspring will be affected by landscape type, with individuals from modern landscapes showing shorter developmental times and higher larval growth rates and pupal mass, as populations in fragmented landscapes may be subject to selection favoring shorter life spans or accelerated development (Duplouy et al., 2013). We further hypothesize that (H2) both field‐caught females and their offspring from modern as compared with traditional agricultural landscapes will exhibit phenotypes enhancing dispersal ability (e.g., having larger wings and higher thorax–abdomen ratios), assuming genetic adaptation to modern, more fragmented landscapes. We predict that the patterns of responses of offspring to landscape parameters will be consistent with those found in their (field‐caught) mothers, indicating a heritable component. Also, we predict that (H3) flight capacity, which is arguably closely linked to dispersal ability, but not propensity will be enhanced in butterfly offspring from modern landscapes. Flight propensity, in contrast, should be affected by adult feeding treatment and thereby food availability. Hence, we predict that (H4) food‐restricted butterflies are more willing to fly. Finally, we expect (H5) butterfly condition to differ across landscapes in field‐caught females but not in their offspring. This is because field‐caught females from modern landscapes might be exposed to reduced nectar availability, while in the offspring, such detrimental effects are controlled for.

## MATERIALS AND METHODS

### Study organism

As study organism, we used the Small Heath, *Coenonympha pamphilus* (Satyrinae), which is a widespread butterfly in the Western Palearctic (Kudrna, 2002). Typically, the species has two generations per year, flying from May to early September (Wickman et al., 1990). The larvae of *C. pamphilus* feed mainly on Poaceae species, undergo four larval instars, and overwinter in the third instar; adults feed on a variety of nectar plants (Ebert & Rennwald, 1991; Roos, 1978; Tolman & Lewington, 1997). The species prefers grassland habitats such as unfertilized pastures, meadows and others, and is one of the most abundant species in such habitats (van Swaay et al., 2008; Windig et al., 2000). Due to its rather sedentary nature, *C. pamphilus* is considered to have a moderate degree of mobility (Pollard & Yates, 1995). Over the long term (1971/1976–2019), a 49% population decline and a 67% decline in distribution in the UK have been observed (Fox et al., 2022), likely caused by agricultural intensification and habitat loss (Polus et al., 2007). However, trends from Germany indicate relatively stable populations between 2006 and 2020 (Kühn et al., 2021).

### Study area

**FIGURE 1:**
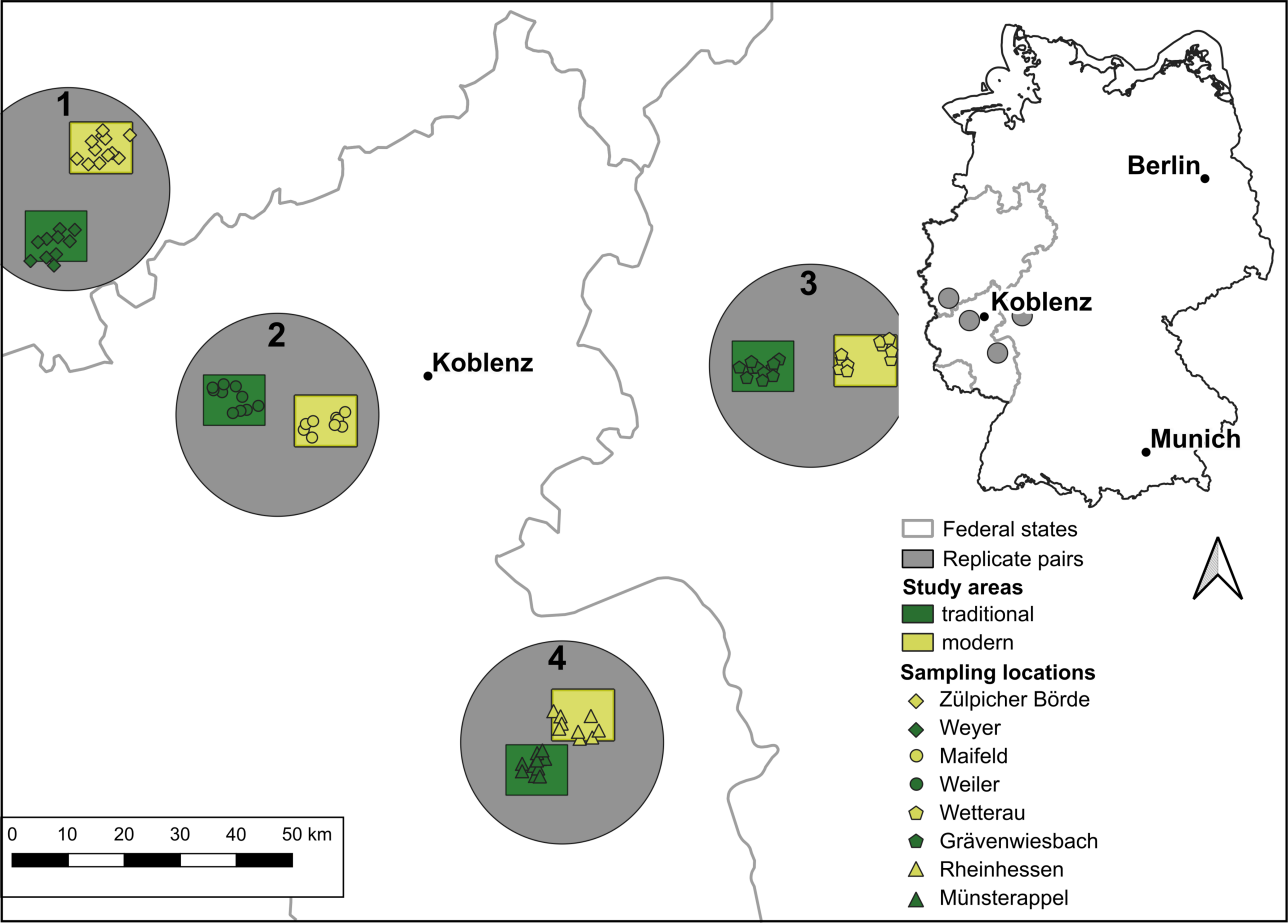
Sampling locations in modern (yellow symbols) and traditional landscapes (green symbols) within the eight study areas (9 × 11 km) and the four replicate pairs (gray shading). The inset shows the location of the study areas in the federal states of Rhineland‐Palatinate, Hesse, and North Rhine‐Westphalia, Germany. The map was created using QGIS version 3.22 (QGIS.org, 2024).

We used four pairs of landscapes, consisting of a “modern” and a “traditional” landscape each (replicate pair 1: “Zülpicher Börde” and “Weyer”; replicate pair 2: “Maifeld” and “Weiler”; replicate pair 3: “Wetterau” and “Grävenwiesbach”; and replicate pair 4: “Rheinhessen” and “Münsterappel”). All eight landscapes were approximately 11 × 9 km in size and were located in the mid‐west of Germany (Figure 1). Modern and traditional landscapes differed in their composition and configuration (within each of the investigated scales: 250‐, 500‐, and 2000‐m radius around the sampling locations), in particular in the proportion of crop fields, grassland, and forests. The modern landscapes are characterized by high proportions of crop fields, low proportions of grassland and forest, whereas the traditional landscapes are characterized by lower proportions of crop fields and higher proportions of grassland and forest (Appendix S1: Table S1). Modern and traditional landscapes also differed regarding their configuration, for example (in the 2000‐m radius around sampling locations) in the mean size of crop fields (modern: 5.1 ha, traditional: 2.7 ha) and the mean size of grassland patches (modern: 0.8 ha, traditional: 1.5 ha). However, we acknowledge that our study does not include data on agrochemical use, which is a limitation in assessing the full extent of agricultural intensification in the study areas. The mean annual precipitation (2013–2022) is 564 mm per year and the mean temperature (2013–2022), 11.1°C for replicate pair 1, 544 mm and 11.3°C for replicate pair 2, 572 mm and 11.2°C for replicate pair 3, and 592 mm and 10.5°C for replicate pair 4 (German Weather Service, 2021).

### Data collection

To investigate variation in developmental traits, condition, morphology, and flight performance, 38–43 females per landscape were sampled at 9–12 locations each, resulting in a total of 82 sampling locations (Appendix S1: Table S2). We sampled between 1 and 10 (4 on average) individuals per location, that is, 325 females in total. All butterflies were sampled between May and August 2022 or between May and August 2023 within their habitat, that is, grassland patches. The field‐caught females were transferred to the laboratory and kept in a climate cabinet with a constant temperature of 26°C and a photoperiod of L18:D6. Females were placed individually in translucent plastic pots (1 L) covered with gauze. Each individual was provided with fresh flowers, freshly cut leaves of host plants (*Poa annua* and *Festuca rubra*), and a highly concentrated sucrose solution. Eggs were collected daily and transferred, separated by female, to small glass vials, which were kept at a temperature cycle mimicking summer temperatures (5 h: 17°C, 5 h: 20°C, 2 h: 22°C, 7 h: 27°C, 3 h: 24°C, 2 h: 20°C) and a L18:D6 photoperiod until hatching (Panasonic MIR‐554, Memmert HPP). After egg‐laying, females were preserved for further analyses by freezing them at −80°C.

After hatching, larvae were placed into transparent plastic boxes (250 mL) with moistened filter paper and fresh cuttings of *P. annua* and *F. rubra*. The boxes were cleaned and provided with new host plants daily. Light and temperature conditions remained unchanged. Potential differences in temperature and light within the climate cabinet were minimized by shifting the location of the boxes on a daily basis. After pupation, pupae were weighed and placed in a new plastic box with moistened filter paper. They were sprayed daily with water until butterfly eclosion. Egg development time, larval time, pupal time, pupal mass, and larval growth rate (mean mass gain per day, natural logarithm of pupal mass divided by larval time) were scored for each individual, to test for heritable variation in developmental traits and thus life history strategies (Nylin & Gotthard, 1998). Upon adult eclosion, butterflies were randomly assigned to two different feeding treatments: ad libitum feeding, where the butterflies had continuous access to water and nectar in the form of flowering plants; or restricted feeding, where the butterflies had access to water and nectar only on the first day, followed by 2 days of access to water only. On day 4 of adult life, butterflies were subjected to the flight tests outlined below.

A flight propensity test (Reim, Blesinger, et al., 2018b) was performed using cages (50 × 50 × 50 cm) equipped with LED‐lights with UVA, being placed in a climate cabinet with a constant temperature of 27°C. All butterflies of the same eclosion day were tested together. They were individually numbered on their hindwing and then placed in the back‐right corner of the cages, after which lights were switched on. In the opposite corner, we placed flowering plants as a nectar source (e.g., *Achillea millefolium*, *Alliaria petiolata*, and *Centaurea jacea*) and leaves of host plants (*P. annua* and *F. rubra*). We scored the time taken to reach the food source for a maximum of 90 min. Flight propensity is given as the time until reaching the food source, with lower values reflecting higher flight propensity. Flight capacity (Ducatez, Legrand, et al., 2012b) was tested by placing butterflies individually in a plastic chamber (30 × 16 × 14 cm) with an opening of 4 cm at the base and placing the chamber on a vortex (IKA Vortex‐Genie 2). Following a 30‐s habituation period, the vortex was switched on for 60 s to strongly shake the chamber, forcing the butterfly to fly, as they would otherwise lay uncomfortably at the chamber's bottom. The duration of flight was measured for each individual, with higher values reflecting higher flight capacity. Subsequently, butterflies were preserved for further analyses by freezing them at −80°C.

Frozen butterflies (field‐caught females and offspring) were dissected by removing the head, legs, and wings with a scalpel. Abdomen and thorax were then separated and weighed to the nearest 0.01 mg. We then calculated the corresponding thorax–abdomen ratio, which serves as an indicator of the relative investment in flight versus reproduction (Guerra, 2011). Left forewings were photographed and the resulting images were used to measure forewing length from the base to the apex of the forewing and forewing area, both as indicators for long distance flights and thus dispersal ability (Altizer & Davis, 2010; Flockhart et al., 2017). In addition, we calculated wing loading (total body mass divided by forewing area) as a predictor of acceleration capacity and flight velocity (Berwaerts et al., 2002; Betts & Wootton, 1988; Danthanarayana, 1976), and wing aspect ratio (four times forewing length^2^ divided by forewing area; Berwaerts et al., 2002) as an indicator of wing shape and hence aerodynamics of flight (Dudley, 2000; Le Roy et al., 2019). Abdomen fat content, as an indicator of storage reserves (Arrese & Soulages, 2010), was determined following Fischer et al. (2003). Briefly, abdomens were dried at 60°C for 24 h, and the initial dry mass was recorded. Then, fat extraction was performed twice for 48 h, each using 1.6 mL of dichloromethane (CH_2_Cl_2_). Afterwards, abdomens were dried again for 24 h and reweighed to determine the fat‐free dry mass. Relative fat content was determined by dividing the total fat content (mass difference between initial dry mass and fat‐free dry mass) by abdomen mass × 100.

### Landscape parameters

To test for effects of landscape parameters on butterfly traits, we distinguished 15 landcover types based on digital orthophotos from 2021 (replicate pairs 1–3) or 2022 (replicate pair 4). Landcover types consisted of crop fields, grassland, fallow, grassy field/road margin, forest (>1 ha), woodland (<1 ha, including hedges, shrubs, and tree lines), orchard, water body, settlement, road, vineyard, tree nursery, fruit plantation, solar panels, and “other land cover types,” for example, gravel pits. We assessed the compositional landscape heterogeneity (diversity of habitat types) by calculating the Shannon diversity index of all 15 landcover types according to Perović et al. (2015). To test for characteristics indicative of modern or traditional agricultural landscapes, we included crop fields, grassland, fallow, grassy field/road margin, forest, woodland, and orchard in subsequent analyses. However, we merged the categories fallow with grassy field/road margin as well as woodland with orchards due to very low coverage of some of these landcover types. The proportion of each of the resulting landcover type, and the patch area and perimeter for crop fields and grassland were determined using QGIS 3.22 (QGIS.org, 2024). The configurational landscape heterogeneity was determined by using the means of the perimeter‐to‐area ratio of crop fields and grassland patches for each sampling location, with higher values indicating increased boundary length and thus heterogeneity for each sampling location. In addition, we determined mean nearest distance among grassland patches, distance between sampling location and nearest grassland habitat patch, and patch size of sampling location. Landscape parameters were determined across three different spatial scales, with radii of 250, 500, and 2000 m around sampling locations except for the parameters distance between sampling location and nearest grassland habitat patch, and patch size of sampling location, as they do not differ between radii. The radii were chosen to test for effects of the immediate habitat surrounding, capturing essential resources utilized for daily activities (250 m), to test for the effects of a broader area, reflective of the typical movement range of *Coenonympha pamphilus* to offer insights into how more distant landscape features and patch connectivity affect trait variation (500 m), and finally to test on a larger landscape scale to account for potential dispersal distances to understand large‐scale effects of habitat fragmentation and the potential for butterflies to move across fragmented habitats.

### Statistical analyses

To test for the effect of landscape type on developmental traits (H1), morphological traits (H2), flight performance (H3, H4), and condition (H5) of offspring, we performed general linear mixed models with landscape type (modern/traditional), sampling year, generation (first/second) nested in sampling year, sex, and the interaction between landscape type and sex as fixed factors. We also included replicate landscape pair (nested within landscape type), sampling location (nested within landscape type and replicate pair), and family (i.e., all offspring produced by an individual female; nested within landscape type, replicate pair, and location) as random factors. For morphological (adult offspring) traits and flight performance, we additionally incorporated feeding treatment, the interaction between feeding treatment and sex, the interaction between feeding treatment and landscape type, and the three‐way interaction between these factors as fixed factors. To meet the requirements for general linear mixed models, we used the *bestNormalize* function (bestNormalize package, Peterson & Cavanaugh, 2020, Peterson, 2021) to transform our trait data prior to analyses (Appendix S1: Table S3). For pairwise comparisons, Tukey HSD post hoc tests were performed.

To test for the effects of landscape type on morphological traits (H2) and condition (H5) of field‐caught females, we also performed general linear mixed models with landscape type, sampling year, and generation nested within sampling year as fixed factor, and replicated landscape pairs (nested within landscape type) and sampling location (nested within landscape type and replicate pair) as random factors.

To investigate the effects of landscape parameters (hypotheses H1–H3, H5), we first ran pairwise correlation analyses among the 11 landscape parameters (Appendix S1: Table S4). In case of strongly correlated parameters (*r* > |0.7|), we removed the one with the higher sum of correlation coefficients. Afterwards, models were built for each response variable (i.e., developmental or morphological traits, flight performance, condition) and all remaining landscape parameters (Appendix S1: Table S4) as well as year and generation nested within years as fixed factors and replicate pair, location, and family (for offspring data) as random factors. However, random factors were only kept if their variance was >0.05 to avoid singularity issues (*VarCorr* function, lme4 package, Bates et al., 2015). We standardized the landscape parameters using the *scale* function. For model selection, we used the *dredge* function (MuMln package, Bartoń, 2024) and selected the top‐ranked models within ΔAICc ≤2. We then generated averaged parameter estimates from this set of models using the *model.avg* function (MuMln package, Bartoń, 2024). If only one model was fitting to this criterion, we used the *lmer* function (lme4 package, Bates et al., 2015) to calculate the model. Analyses were performed separately for each landscape radius and sex to account for the different ecological and behavioral traits exhibited by males and females. All statistical tests were conducted using R 4.4.2 (R Core Team, 2024).

## RESULTS

In total, we analyzed 973 offspring from 147 field‐caught females. Included females originated from 61 different sampling locations (4–10 sampling locations per landscape, 7.6 on average). Females produced 1–28 (on average 6.6) offspring, resulting in 64–239 offspring per landscape (on average 116 offspring per landscape). All traits, except wing aspect ratio and egg development time, differed significantly between sexes, indicating that larval time, masses (pupal, abdomen, thorax), forewing length, forewing area, and wing loading were higher in females compared to males. In contrast, pupal time, larval growth rate, thorax–abdomen ratio, relative fat content, and flight propensity and capacity were higher in males compared to females (Appendix S1: Table S5).

### Developmental traits of offspring (H1)

**FIGURE 2:**
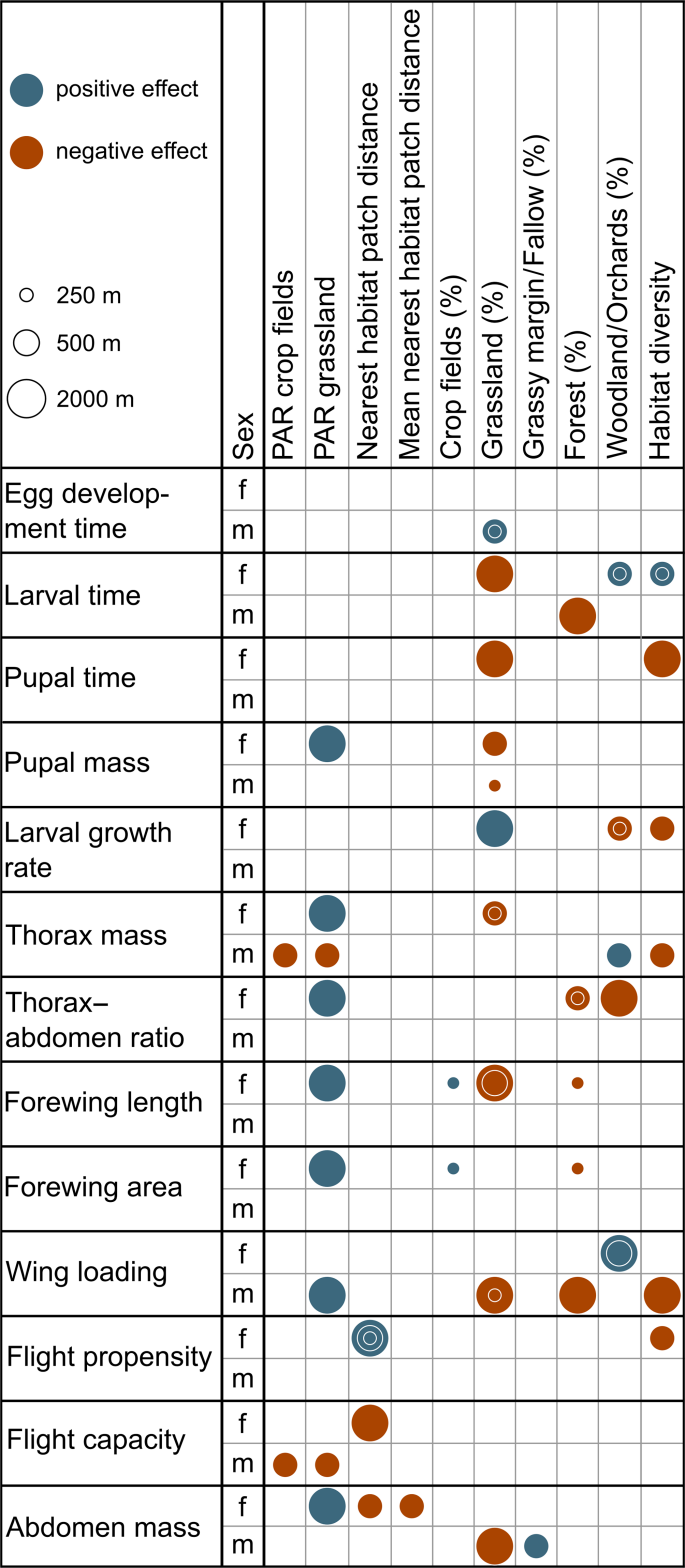
Summary of the significant effects of landscape parameters measured in three different radii around the sampling locations on various traits in offspring of *Coenonympha pamphilus*. Shown are significant positive (blue) and negative (red) effects of 10 out of 11 landscape parameters (sampling location patch size showed no effect on any traits) on female (f) and male (m) traits. Different‐sized circles indicate the effects within the different radii. See Appendix S1: Table S4 for the parameters excluded from the analysis due to high correlation with others. PAR, perimeter‐to‐area ratio.

None of the developmental traits measured were significantly affected by the factor landscape type (Table 1). Thus, egg development time, larval time, pupal time, pupal mass, and larval growth rate did not differ between modern and traditional landscapes (Appendix S1: Table S6). However, for at least one sex, one or more significant landscape parameters were found for all developmental traits (Figure 2, Appendix S1: Table S7). In males, egg development time was positively affected by the proportion of grassland (250‐ and 500‐m radius). Thus, egg development was faster in areas with a lower proportion of grassland. In females, larval time was negatively related to the proportion of grassland in the 2000‐m radius, indicating faster development with higher amounts of grassland. Female larval time was additionally positively related to the habitat diversity at all three spatial scales and to the proportion of woodland/orchards (250‐ and 500‐m radius). Hence, larval development of females was faster in landscapes with lower amounts of woodland/orchards and low habitat diversity. In males, larval time was negatively affected by the proportion of forest (2000‐m radius), indicating faster development with increasing proportion of forest.

**TABLE 1:** Results of general linear mixed models for the effects of the fixed factors landscape type (LST), sex, LST × sex interaction, year, and generation (nested within year) on various traits measured in the offspring of *Coenonympha pamphilus*.

Trait/factor	Estimate	SE	*t* value	*p*
Egg development time				
LST	0.210	0.15	1.43	0.1553
Sex	0.123	0.08	1.60	0.1101
LST × sex	−0.171	0.11	−1.49	0.1370
Year	0.197	0.14	1.37	0.1730
Generation (within year 1)	0.155	0.20	0.79	0.4300
Generation (within year 2)	−0.415	0.22	−1.84	0.0664
Larval time				
LST	−0.105	0.17	−0.61	>0.9999
Sex	0.624	0.06	10.75	**<0.0001**
LST × sex	0.048	0.09	0.55	0.5809
Year	1.122	0.10	10.83	**<0.0001**
Generation (within year 1)	0.418	0.15	2.79	**0.0064**
Generation (within year 2)	0.884	0.16	5.39	**<0.0001**
Pupal time				
LST	0.007	0.14	0.05	0.9621
Sex	−0.258	0.08	−3.32	**0.0009**
LST × sex	0.022	0.12	0.19	0.8487
Year	−0.068	0.12	−0.57	0.5689
Generation (within year 1)	−0.347	0.17	−2.03	**0.0469**
Generation (within year 2)	−0.399	0.21	−1.95	**0.0520**
Pupal mass				
LST	−0.189	0.19	−1.00	>0.9999
Sex	1.276	0.06	21.85	**<0.0001**
LST × sex	−0.112	0.09	−1.29	0.1993
Year	−0.051	0.09	−0.56	0.5769
Generation (within year 1)	−0.683	0.14	−4.91	**<0.0001**
Generation (within year 2)	−0.433	0.16	−2.79	**0.0057**
Larval growth rate				
LST	0.050	0.15	8.28	>0.9999
Sex	−0.344	0.06	881.53	**<0.0001**
LST × sex	−0.048	0.09	884.31	0.5838
Year	−1.164	0.11	107.30	**<0.0001**
Generation (within year 1)	−0.632	0.16	82.61	**0.0001**
Generation (within year 2)	−1.013	0.17	318.56	**<0.0001**
Thorax mass				
LST	−0.261	0.26	−1.02	0.3104
Sex	1.123	0.10	11.57	**<0.0001**
LST × sex	−0.045	0.14	−0.32	0.7525
Year	−0.280	0.08	−3.58	**0.0006**
Generation (within year 1)	−0.443	0.12	−3.77	**0.0004**
Generation (within year 2)	−0.214	0.16	−1.38	0.1687
Feeding treatment	−0.076	0.09	−0.85	0.3946
LST × feeding treatment	0.071	0.13	0.53	0.5963
Sex × feeding treatment	0.146	0.13	1.11	0.2695
LST × sex × feeding treatment	−0.050	0.20	−0.26	0.7965
Thorax–abdomen ratio				
LST	−0.012	0.09	−0.12	>0.9999
Sex	−1.569	0.08	−20.26	**<0.0001**
LST × sex	−0.060	0.11	−0.53	0.5956
Year	0.533	0.05	11.70	**<0.0001**
Generation (within year 1)	0.245	0.07	3.69	**0.0004**
Generation (within year 2)	0.036	0.11	0.32	0.7521
Feeding treatment	0.018	0.07	0.25	0.8033
LST × feeding treatment	−0.083	0.11	−0.78	0.4338
Sex × feeding treatment	−0.005	0.11	−0.05	0.9633
LST × sex × feeding treatment	0.112	0.16	0.73	0.4688
Forewing length				
LST	−0.157	0.25	−0.63	0.5324
Sex	0.858	0.10	8.74	**<0.0001**
LST × sex	−0.217	0.14	−1.50	0.1342
Year	0.359	0.09	3.87	**0.0002**
Generation (within year 1)	−0.717	0.14	−5.24	**<0.0001**
Generation (within year 2)	−0.871	0.17	−5.02	**<0.0001**
Feeding treatment	−0.094	0.09	−1.05	0.2933
LST × feeding treatment	0.017	0.13	0.13	0.8967
Sex × feeding treatment	0.146	0.13	1.09	0.2745
LST × sex × feeding treatment	0.151	0.20	0.76	0.4461
Forewing area				
LST	−0.176	0.22	−0.80	0.4280
Sex	0.893	0.10	8.93	**<0.0001**
LST × sex	−0.169	0.15	−1.14	0.2540
Year	−0.048	0.10	−0.46	0.6450
Generation (within year 1)	−0.853	0.15	−5.61	**<0.0001**
Generation (within year 2)	−0.945	0.18	−5.13	**<0.0001**
Feeding treatment	−0.144	0.09	−1.57	0.1170
LST × feeding treatment	0.084	0.14	0.61	0.5410
Sex × feeding treatment	0.190	0.14	1.40	0.1620
LST × sex × feeding treatment	0.066	0.20	0.33	0.7450
Wing loading				
LST	−0.288	0.12	−2.34	0.0329
Sex	1.395	0.08	18.03	**<0.0001**
LST × sex	0.181	0.11	1.59	0.1119
Year	−0.524	0.06	−9.05	**<0.0001**
Generation (within year 1)	−0.181	0.09	−2.05	**0.0441**
Generation (within year 2)	0.106	0.13	0.85	0.3978
Feeding treatment	−0.079	0.07	−1.12	0.2648
LST × feeding treatment	0.123	0.11	1.16	0.2448
Sex × feeding treatment	0.057	0.10	0.54	0.5906
LST × sex × feeding treatment	−0.101	0.16	−0.65	0.5156
Wing aspect ratio				
LST	−0.052	0.23	−0.23	>0.9999
Sex	0.128	0.11	1.12	0.2630
LST × sex	−0.106	0.17	−0.63	0.5280
Year	0.946	0.08	11.46	**<0.0001**
Generation (within year 1)	0.096	0.12	0.81	0.4200
Generation (within year 2)	−0.016	0.19	−0.09	0.9310
Feeding treatment	0.050	0.10	0.48	0.6350
LST × feeding treatment	−0.071	0.16	−0.45	0.6500
Sex × feeding treatment	−0.023	0.15	−0.15	0.8800
LST × sex × feeding treatment	0.134	0.23	0.58	0.5600
Abdomen mass				
LST	−0.253	0.10	−2.41	>0.9999
Sex	1.450	0.07	20.74	**<0.0001**
LST × sex	0.177	0.10	1.72	0.0858
Year	−0.609	0.05	−11.34	**<0.0001**
Generation (within year 1)	−0.392	0.08	−4.81	**<0.0001**
Generation (within year 2)	−0.072	0.11	−0.66	0.5126
Feeding treatment	−0.122	0.06	−1.89	0.0588
LST × feeding treatment	0.187	0.10	1.95	0.0519
Sex × feeding treatment	0.172	0.10	1.81	0.0713
LST × sex × feeding treatment	−0.203	0.14	−1.44	0.1502
Relative fat content				
LST	0.027	0.12	0.23	0.8160
Sex	−0.778	0.10	−7.57	**<0.0001**
LST × sex	−0.106	0.15	−0.70	0.4841
Year	−1.006	0.07	−13.62	**<0.0001**
Generation (within year 1)	−0.914	0.11	−8.67	**<0.0001**
Generation (within year 2)	−0.501	0.16	−3.16	**0.0017**
Feeding treatment	0.014	0.09	0.15	0.8847
LST × feeding treatment	−0.064	0.14	−0.45	0.6510
Sex × feeding treatment	−0.049	0.14	−0.35	0.7241
LST × sex × feeding treatment	0.215	0.21	1.04	0.2973
Flight propensity				
LST	0.083	0.13	0.66	>0.9999
Sex	−0.277	0.11	−2.44	**0.0148**
LST × sex	−0.191	0.17	−1.13	0.2605
Year	0.178	0.06	2.74	**0.0063**
Generation (within year 1)	0.244	0.09	2.62	**0.0090**
Feeding treatment	−0.288	0.11	−2.71	**0.0069**
LST × feeding treatment	0.002	0.16	0.01	0.9925
Sex × feeding treatment	0.427	0.16	2.72	**0.0068**
LST × sex × feeding treatment	−0.127	0.23	−0.54	0.5863
Flight capacity				
LST	−0.115	0.17	−0.69	0.4884
Sex	−0.426	0.15	−2.93	**0.0035**
LST × sex	−0.019	0.22	−0.09	0.9304
Year	−0.026	0.09	−0.29	0.7761
Generation (within year 1)	−0.549	0.13	−4.25	**<0.0001**
Feeding treatment	−0.067	0.14	−0.49	0.6253
LST × feeding treatment	0.077	0.21	0.37	0.7119
Sex × feeding treatment	0.108	0.20	0.53	0.5944
LST × sex × feeding treatment	−0.085	0.30	−0.28	0.7774

*Note*: Significant *p* values are given in bold. Results on adult traits additionally include the fixed factor feeding treatment and its interactions. Random factors included in the models were replicate pair (nested within LST), sampling location (nested within LST and replicate pair), and family (nested within LST, replicate pair and location).

Female pupal time was negatively affected by habitat diversity (2000‐m radius), suggesting faster pupal development in areas with higher habitat diversity. Female pupal time was also negatively impacted by the proportion of grassland (2000‐m radius), suggesting faster development with higher proportions of grassland at the landscape scale. Female pupal mass was positively affected by the perimeter‐to‐area ratio of grassland in the 2000‐m radius, but negatively by the proportion of grassland (500‐m radius). Similarly, male pupal mass was negatively affected by the proportion of grassland in the 250‐m radius. Female larval growth rate was negatively affected by the habitat diversity in the 500‐m radius, indicating a higher daily mass gain in areas with less diverse landscapes. Also, the larval growth rate of females was negatively affected by the proportion of woodland/orchards (250‐ and 500‐m radius) and positively by the proportion of grassland (2000‐m radius).

Across all three radii and sexes, the magnitude of standardized effects, that is, estimates of standardized landscape parameters, varied between |β| = 0.112 ± 0.056 and 0.229 ± 0.06 (Appendix S1: Tables S7 and S9, Figure S1). Regarding individual traits, pupal mass (mean |β| = 0.187 ± 0.074) and egg development time (|β| = 0.171 ± 0.073) exhibited the largest landscape‐related responses, whereas larval growth rate had the smallest mean response (mean |β| = 0.138 ± 0.058). Among individual landscape parameters, the strongest effects were associated with the perimeter‐to‐area ratio of grassland (|β| = 0.229 ± 0.06). In contrast, the proportion of forests showed the weakest average effects (|β| = 0.112 ± 0.056).

### Morphological traits of field‐caught butterflies and their offspring (H2)

Landscape type did not significantly affect any of the dispersal‐related morphological traits measured in field‐caught females (Table 2) or their offspring (Table 1). Thus, animals from modern compared with traditional agricultural landscapes did not differ in thorax, thorax–abdomen ratio, forewing length, forewing area, wing loading, and wing aspect ratio (Appendix S1: Tables S6 and S8). However, at least one significant landscape parameter in at least one radius was found for all morphological traits (Figure 2, Appendix S1: Tables S7 and S9). In field‐caught females, thorax mass was positively affected by the perimeter‐to‐area ratio of crop fields (500‐m radius), indicating that longer boundaries and smaller crop fields were associated with heavier thoraxes. The thorax–abdomen ratio was positively affected by the proportion of grassy margins/fallows (250‐ and 2000‐m radius), suggesting relatively heavier thoraxes in surroundings with more grassy margins/fallows. Forewing area was negatively affected by the proportion of grassland (2000‐m radius), but positively with the proportion of grassy margins/fallows (2000‐m radius). Wing loading was positively affected by the perimeter‐to‐area ratio of crop fields and the proportion of grassland in the 500‐m radius. Wing aspect ratio was positively affected by the perimeter‐to‐area ratio of grassland (2000‐m radius, Appendix S1: Table S9).

**TABLE 2:** Results of general linear mixed models for the effects of the fixed factors landscape type (LST), year, and generation (nested within year) on various traits in field‐caught females of *Coenonympha pamphilus*.

Trait/factor	Estimate	SE	*t* value	*p*
Thorax mass				
LST	0.042	0.15	0.28	>0.9999
Year	−1.050	0.18	−5.84	**<0.0001**
Generation (within year 1)	−0.716	0.25	−2.81	**0.0056**
Generation (within year 2)	0.104	0.22	0.48	0.6322
Thorax–abdomen ratio				
LST	−0.098	0.17	−0.57	0.5677
Year	0.642	0.19	3.37	**0.0010**
Generation (within year 1)	0.451	0.27	1.67	0.0969
Generation (within year 2)	0.263	0.23	1.14	0.2562
Forewing length				
LST	−0.072	0.25	−0.29	0.7782
Year	−0.303	0.20	−1.53	0.1276
Generation (within year 1)	−0.774	0.30	−2.62	**0.0119**
Generation (within year 2)	−0.061	0.24	−0.26	0.7982
Forewing area				
LST	−0.159	0.38	−0.42	0.6766
Year	−0.611	0.19	−3.30	**0.0013**
Generation (within year 1)	−1.016	0.30	−3.42	**0.0010**
Generation (within year 2)	−0.068	0.23	−0.30	0.7661
Wing loading				
LST	0.191	0.19	0.99	0.3242
Year	−1.153	0.18	−6.54	**<0.0001**
Generation (within year 1)	−0.650	0.25	−2.61	**0.0100**
Generation (within year 2)	0.132	0.21	0.62	0.5336
Wing aspect ratio				
LST	0.292	0.52	0.57	0.5734
Year	0.780	0.19	4.18	**0.0001**
Generation (within year 1)	0.616	0.27	2.31	**0.0231**
Generation (within year 2)	0.049	0.23	0.22	0.8299
Abdomen mass				
LST	0.371	0.85	0.44	>0.9999
Year	−7.794	1.00	−7.82	**<0.0001**
Generation (within year 1)	−5.542	1.41	−3.93	**0.0001**
Generation (within year 2)	0.017	1.20	0.01	0.9886
Relative fat content				
LST	0.090	0.16	0.56	0.6010
Year	−0.666	0.18	−3.65	**0.0004**
Generation (within year 1)	−0.714	0.26	−2.74	**0.0098**
Generation (within year 2)	−0.508	0.22	−2.30	**0.0232**

*Note*: Significant *p* values are given in bold. Random factors included in the models were replicate pair (nested within LST) and sampling location (nested within LST and replicate pair).

In female offspring, thorax mass was positively related to the perimeter‐to‐area ratio of grassland (2000‐m radius), and negatively to the proportion of grassland (250‐ and 500‐m radius). In male offspring, thorax mass was positively related to the proportion of woodland/orchards and negatively to habitat diversity and perimeter‐to‐area ratio of both crop fields and grassland (500‐m radius). The thorax–abdomen ratio of female offspring was positively affected by perimeter‐to‐area ratio of grassland (2000‐m radius) and negatively by the proportion of forest (250‐ and 500‐m radius) and the proportions of woodland/orchards (2000‐m radius). In female offspring, forewing length and forewing area were both positively affected by the proportion of crop fields and negatively by the proportion of forest (250‐m radius). In addition, female forewing length was positively related to the perimeter‐to‐area ratio of grassland (2000‐m radius) and negatively to the proportion of grassland (500‐ and 2000‐m radius). Thus, females occurred with more heterogeneous grassland patches and higher grassland proportions. Wing loading was positively affected by the proportion of woodland (500‐ and 2000‐m radius) in female offspring. In male offspring, it was positively affected by the perimeter‐to‐area ratio of grassland (2000‐m radius), but negatively by the proportions of grassland (250‐ and 2000‐m radius) and forest (200‐m radius) and by habitat diversity (2000‐m radius; Appendix S1: Table S7).

Dispersal‐related morphological traits showed responses to landscape parameters across sexes and spatial scales between |β| = 0.089 ± 0.044 and 0.270 ± 0.092 (Appendix S1: Tables S7 and S9, Figure S1). Wing aspect ratio (field‐caught females: |β| = 0.270 ± 0.092) and thorax mass (mean |β| = 0.179 ± 0.064) exhibited the largest landscape‐related responses, particularly in relation to grassland and crop field configuration. Among the landscape parameters representing compositional and configurational landscape heterogeneity, the strongest mean effects were found for the perimeter‐to‐area ratio of crop fields (mean |β| = 0.186 ± 0.068), the perimeter‐to‐area ratio of grassland (mean |β| = 0.176 ± 0.060), and the proportion of grassy margins/fallows (mean |β| = 0.176 ± 0.084). In contrast, forest cover showed the weakest average effects (mean |β| = 0.117 ± 0.051).

### Flight performance of offspring (H3 and H4)

Flight propensity did not differ significantly between landscape types, but between adult feeding treatments (ad libitum: 12.92 ± 25.78, restricted: 16.98 ± 28.89 min; mean ± SE). A significant feeding treatment by sex interaction indicates that only males had a significantly higher flight propensity when fed restricted (Tukey HSD: ad libitum: 12.6 ± 25.95, restricted: 23.57 ± 32.64 min). Flight capacity also did not differ between landscape types, and additionally not between feeding treatments (Table 1, Appendix S1: Table S6). However, female flight propensity and male flight capacity were significantly affected by landscape parameters (Figure 2, Appendix S1: Table S7). Female flight propensity was positively affected by the nearest habitat patch distance (250‐, 500‐, and 2000‐m radius), indicating a higher propensity in landscapes with less connected grassland habitats. The flight capacity of females was negatively related to the nearest habitat patch distance (2000‐m radius). Male flight capacity was negatively affected by the perimeter‐to‐area ratios of crop fields and grassland in the 500‐m radius.

Across all flight performance models, the strongest mean effect was found for the distance to the nearest habitat patch (mean |β| = 0.153 ± 0.063), followed by the perimeter‐to‐area ratio of grassland (|β| = 0.146 ± 0.054) and habitat diversity (|β| = 0.142 ± 0.072; Appendix S1: Tables S7 and S9, Figure S1). The perimeter‐to‐area ratio of crop fields (|β| = 0.140 ± 0.07) showed the weakest effect. Among individual traits, flight propensity (mean |β| = 0.148 ± 0.064) was slightly strongly affected by the landscape parameters than flight capacity (mean |β| = 0.143 ± 0.062).

### Condition of field‐caught butterflies and their offspring (H5)

Landscape type did not significantly affect the condition measured in field‐caught females (Table 2) or their offspring (Table 1). Thus, modern compared with traditional agricultural landscapes did not differ in relative fat content or abdomen mass (Appendix S1: Tables S6 and S8). Relative fat content of field‐caught females was, however, positively affected by the proportion of woodland and orchards (250‐, 500‐, and 2000‐m radius), and negatively by the proportion of grassy margins/fallows (500‐m radius). The abdomen mass in field‐caught females was positively affected by the proportion of grassland and the perimeter‐to‐area ratio of crop fields in the 500‐m radius, indicating heavier abdomen in landscapes with more grassland and smaller crop fields (Appendix S1: Table S9). Abdomen mass in female offspring was positively related to the perimeter‐to‐area ratio of grassland (2000‐m radius) and negatively to the nearest habitat patch and the nearest distance among grassland patches (500‐m radius). The abdomen mass of male offspring, finally, was positively related to the proportion of grassy margins/fallows (500‐m radius) and negatively to the proportion of grassland (2000‐m radius; Figure 2, Appendix S1: Table S7).

The proportion of woodland/orchards (mean |β| = 0.254 ± 0.088) showed the strongest effect among all landscape parameters and the mean nearest distance among grassland patches, the weakest (|β| = 0.098 ± 0.047; Appendix S1: Tables S7 and S9, Figure S1). Relative fat content (of field‐caught females; mean |β| = 0.247 ± 0.086) was more affected by landscape parameters than abdomen mass (mean |β| = 0.129 ± 0.058).

## DISCUSSION

In this study, we could not detect effects of landscape type per se on morphological traits or condition in field‐caught females of *Coenonympha pamphilus*, and also not on developmental traits, morphology, condition, and flight performance of their offspring. However, most traits were significantly affected by landscape parameters (see below), suggesting that differences in compositional and configurational landscape heterogeneity do have an impact on trait variation. All 11 landscape parameters tested affected traits of *C*. *pamphilus* significantly, except the size of the sampling location, indicating an adequate sampling design.

### Developmental traits of offspring (H1)

Our results indicate, in accordance with our first hypothesis, effects of landscape heterogeneity on developmental traits in *C. pamphilus*. However, we could not clearly work out whether landscapes with low compositional and configurational heterogeneity result in faster or slower development. Egg and larval time decreased, and larval growth rates increased with parameters indicative of agricultural intensification, that is, lower proportions of grassland or woodland/orchards, and low habitat diversity. This might result, for instance, from increased predation, higher habitat instability, or resource scarcity in more intensively used landscapes (Dennis et al., 2006; McCoshum et al., 2016), which may induce higher mortality and thus select for rapid development and early reproduction (Clancy & Price, 1987; Klockmann & Fischer, 2017; Sibly & Calow, 1986). Modern, more fragmented landscapes often exhibit different predator dynamics (Ryall & Fahrig, 2006) compared to traditional, less fragmented ones, due to a scarcity of refuges and the concentration of predators in the few remaining habitat patches (Nauta et al., 2022; Priyadarshana et al., 2024). Therefore, spending less time in more vulnerable life stages (egg, larva, and pupa) might be advantageous in more fragmented landscapes (Välimäki & Kaitala, 2007). Furthermore, in intensified, fragmented landscapes, resources may occur less frequently and predictably, additionally selecting for fast development in order to quickly reach the adult stage with higher dispersal capacity (Bubová et al., 2016; Laurance et al., 2007). Yet, development time also decreased and larval growth rate increased with parameters indicative of more traditional landscapes, that is, high proportions of grassland or forests, high habitat diversity, and a low distance to the nearest grassland habitat. This might suggest that landscapes with enhanced heterogeneity, often providing more stable conditions, allow for more efficient growth leading to shorter development times (Oliver et al., 2010).

Our contradictory results on butterfly development, which do not seem to be related to sex or the radius investigated, indicate that effects of landscape heterogeneity do not (yet) show any clear direction. In general, both highly fragmented and continuous landscapes may host high‐quality habitat patches, and the development time of butterflies might be more tightly linked to habitat quality (e.g., host plant availability and quality, microclimate) than to heterogeneity at the landscape level (Greiser et al., 2022; Raharivololoniaina et al., 2021; Rytteri et al., 2021; Wedell et al., 1997).

With regard to pupal mass, parameters indicative of agricultural intensification, namely small grassland patches with higher edge density, and low proportions of grassland, were associated with heavier pupae. Thus, more fragmented landscapes may select for larger body size, which is often related to increased dispersal ability and storage reserves (Bowler & Benton, 2005; Kuussaari et al., 2014; Schultz et al., 2017), both of which may be advantageous in fragmented landscapes. This pattern is further corroborated by the relatively strong effect size of the relationship between pupal mass and the perimeter‐to‐area ratio of grassland, which was the highest among all developmental traits examined.

### Morphological traits of field‐caught butterflies and their offspring (H2)

In accordance with our second hypothesis, landscape parameters indicative of modern landscapes favored more dispersive phenotypes in both field‐caught females and their offspring. In particular, heavier thoraxes, higher thorax–abdomen ratios, and larger forewings were found with lower habitat diversity, lower proportions of grassland, forests and woodland/orchards, and higher proportions of crop fields, larger crop fields with low boundary length, and smaller grassland patches with higher boundary length. Since not only field‐caught females but also their offspring responded to landscape parameters in a similar manner, variation in dispersal‐related traits might be the result of genetic adaptation (Hill et al., 1999). Thus, a high investment in flight muscles (Baguette & Schtickzelle, 2006; Berwaerts et al., 2002; Guerra, 2011), aerodynamically more efficient wings (Dudley, 2000; Flockhart et al., 2017; Le Roy et al., 2019), and hence dispersal ability might be selected for in order to increase survival probability in fragmented landscapes (Riva et al., 2023; Taylor et al., 1993; Thomas et al., 1998) as predicted. The notion of selection on dispersal ability in modern landscapes is further supported by similar patterns found in our previous study on field‐caught individuals of *C*. *pamphilus* (Deppe et al., 2024). We showed that dispersal‐related morphological traits were enhanced in landscapes with high proportions of crop fields, potentially giving them an advantage in these hostile landscapes to search for suitable habitats. However, variation in field‐caught females and their female offspring was related to different landscape parameters (Appendix S1: Table S10), additionally indicating a substantial influence of the environment on the observed traits. Under controlled conditions, effects of landscape parameters became more pronounced, probably because of reduced noise due to the standardized environment. In addition, we cannot rule out an influence of maternal effects on trait variation, such as the condition of the mothers, which may have affected our current results. In general, maternal effects can be an important source of variation (Badyaev & Uller, 2009; Mousseau & Fox, 1998), though evidence regarding butterflies is limited (Moore & Singer, 1987; Reinhold, 2002).

Interestingly, the enhancement of dispersal‐related morphological traits was related to both configurational and compositional landscape heterogeneity. This might indicate that not only the composition but also the configuration of the landscape predicts suitability for dispersal, and may hence act as a filter for less mobile individuals or species in arthropods (Baguette & Van Dyck, 2007; Deppe et al., 2024; Deppe & Fischer, 2023; Priyadarshana et al., 2021, 2024; Ziv & Davidowitz, 2019).

In general, responses to landscape variation may differ among sexes, as is evident in thorax mass in this study. While it was lower in field‐caught females originating from landscapes with larger crop fields and lower edge density, the opposite was the case for male offspring. Furthermore, a low proportion of grassy margins/fallows was associated with a lower, not higher, thorax–abdomen ratio in females. Such differences might be due to sex‐specific reproduction strategies and hence resource allocation (Karlsson, 1995; Karlsson & Wickman, 1990). Females usually prioritize reproductive capacity, resulting in relatively larger abdomen for egg production, whereas males in general benefit more from enhanced flight capability to find mates and defend territories (Boggs et al., 2003; Reim, Blesinger, et al., 2018b). Thus, fecundity selection may be prioritized by females even in fragmented landscapes. Other studies have also demonstrated that thorax mass is only enhanced in the more dispersive sex in fragmented landscapes (Lion et al., 2022; Merckx & Van Dyck, 2006). Similar considerations might apply to the wing aspect ratio of field‐caught females, which decreased in response to landscape parameters indicative of modern landscapes, that is, smaller grassland patches with higher boundary length.

Also, wing loading showed sex‐specific responses to landscape parameters. While female wing loading (both field‐caught and offspring) decreased with parameters indicative of modern landscapes, that is, low proportion of woodland/orchards or grassland and larger crop fields with low edge density, male wing loading increased when originating from such landscapes (low proportion of grassland, forests, low habitat diversity, and small grassland patches with high boundary length). This might be due to different responses to landscape fragmentation and are likely linked to differences in body composition and reproductive roles. Females typically possess a heavier abdomen due to egg production compared to males, which increases body mass and thus wing loading. However, in modern, simplified landscapes with, for example, large crop fields, females might respond toward enhanced dispersal ability, potentially achieved through a reduction in abdomen mass or increase in forewing length and area. In contrast, the increased wing loading of males likely reflects increased investment in thoracic flight musculature to support faster, more directed flight over open areas during mate searching.

In addition, male thorax mass increased rather than decreased in landscapes with high proportions of woodland and/orchards and larger grassland patches with low boundary length, indicating that these landcover types might not act as suitable stepping stones in the landscape and do not promote habitat connectivity in contrast to, for example, grassy road or field margins. This further indicates that the size of grassland patches alone may not adequately reflect their habitat quality and thus cannot ensure that they effectively serve as suitable habitats or dispersal corridors for butterflies (Fischer et al., 2022; Saura et al., 2014; Saura & Rubio, 2010). This is also supported by the effect sizes, indicating the proportion of grassy margins/fallows and the perimeter‐to‐area ratio of crop fields, that is, the configuration of crop fields in the landscape, as the most important predictors for dispersal‐related morphological adaptations of butterflies.

### Flight performance of offspring (H3 and H4)

Our findings also support hypotheses 3 and 4, indicating that flight propensity (at least in males) is more strongly affected by adult feeding treatment than by landscape type. Thus, flight propensity in male offspring was not affected by landscape parameters but was enhanced under food restriction. A higher flight propensity is believed to be associated with more exploratory behavior, that is, a tendency to actively disperse through the surrounding landscape (Bonte et al., 2012). This in turn increases the probability of emigration and the likelihood of finding suitable habitats (Cote et al., 2010; Fraser et al., 2001; Merckx et al., 2003). Our results thus suggest that food shortage, that is, the reduced availability of accessible resources in the landscape, increases the motivation to explore other patches. This may also be the case in modern landscapes, due to more scattered resources and hence higher adult food stress (Lebeau et al., 2016a; Reim, Baguette, et al., 2018a). A high flight propensity was indeed found to facilitate locating scattered resources in flying insects (reviewed in Cote et al., 2017). Alternatively, habitat fragmentation may select for reduced flight propensity due to the more hostile landscape matrix and hence higher costs of dispersal (Bergerot et al., 2012; Deppe et al., 2024; Merckx et al., 2003; Schtickzelle et al., 2006).

However, flight propensity of females was not affected by feeding treatment but was higher when originating from landscapes indicative of modern landscapes, that is, with a high distance to the nearest grassland habitat. This might suggest that exploratory behavior does not only originate from the nutritional status of an individual, but might also indicate that individuals from resource‐poor landscapes show a greater inherent tendency to disperse and explore, potentially as an adaptive response to locate suitable habitats or resources under challenging landscape conditions (Evans et al., 2020; Reim, Baguette, et al., 2018a; Van Dyck & Baguette, 2005). However, it is generally difficult to draw robust conclusions about the dispersal process based on data obtained under artificial laboratory conditions, as is the case here (cf. Stevens et al., 2010).

Flight capacity of offspring was, contrary to our hypothesis and to previous studies (Duplouy et al., 2013; Lebeau et al., 2016a; Merckx et al., 2003; Priyadarshana et al., 2024), not enhanced in landscapes with reduced heterogeneity. This is consistent with our previous results on field‐caught males of *C. pamphilus* (Deppe et al., 2024). One reason for this may be that our flight capacity test, due to its short duration, may not reflect long‐term dispersal, but is rather indicative of dispersal under stressful conditions such as escaping predators or navigating through unsuitable habitats (Ducatez, Baguette, et al., 2012a). However, flight capacity of male offspring was enhanced in landscapes with large grassland patches with low boundary length. This might indicate that lower configurational heterogeneity of grassland patches, that is, less connected habitats and thus resources, enhances flight capacity. As the quality of resources in grassland patches plays an important role in butterfly occurrence (Collinge et al., 2003; Pywell et al., 2004), the size of potential habitat patches might not be a good indicator of their suitability as a habitat. Thus, even in landscapes with large grassland patches and low boundary length, the need to travel greater distances in search of food and mates might select for improved flight skills. This is also supported by an enhanced flight capacity of male offspring in landscapes with large crop fields and lower boundary length. As a result, individuals with higher flight capacity are more likely to thrive, being able to effectively navigate and exploit scattered resources (Dover & Settele, 2009; Öckinger & Van Dyck, 2012; Schtickzelle et al., 2006; Schtickzelle & Baguette, 2003; Thomas, 2000).

Overall, the effect sizes of landscape parameters affecting the flight performance were relatively similar, with considerable overlap in their confidence intervals. This suggests that there are no strong differences in the effects of these parameters on flight performance.

### Condition of field‐caught butterflies and their offspring (H5)

In line with our last hypothesis, the relative fat content of field‐caught females increased with higher proportions of woodland and orchards. This is surprising, as our results above suggested that woodland and orchards do not provide ample food sources and do not serve as stepping stones (cf. Dennis et al., 2013; Dover & Settele, 2009). However, this result fits to our previous study on field‐caught males, which showed increased storage reserves with decreasing distance to the nearest habitat patch (that is, with decreasing isolation), highlighting the importance of suitable habitat in the landscape matrix (Deppe et al., 2024; Lebeau et al., 2016b). In our current study, though, the relative fat content of field‐caught females was enhanced in landscapes with a low proportion of grassy margins and fallows. This strengthens the idea that rather the accessibility, connectivity, or quality of habitat patches is important for the fulfillment of energy requirements of butterflies (Collinge et al., 2003; Jain et al., 2020; Kuussaari et al., 2007; Pywell et al., 2004; van Halder et al., 2017). The relative fat content of offspring, in contrast, did not differ at all between feeding treatments. This is in contrast with other studies having shown that adult food intake influences butterfly condition (Cahenzli & Erhardt, 2012; Lebeau et al., 2016b; Pegram et al., 2013; Reim et al., 2019). Perhaps, the duration of the feeding treatments in our experiment (three days) was insufficient to reveal significant differences in fat storage. Also, variation in larval food intake, which was not completely standardized for all individuals in our study, may have affected storage reserves (Boggs & Freeman, 2005).

However, individuals (field‐caught females or offspring) originating from landscapes with low proportions of grassy margins/fallows or grassland, larger crop fields with lower edge density, and high distances to the nearest habitat and grassland patch showed a lower abdomen mass. Thus, less intensified agricultural landscapes may comprise environments better supporting the accumulation of storage reserves, in line with our findings on development. In contrast, the proportion and size of grassland patches showed the opposite effect, with offspring originating from landscapes with smaller and lower proportions of grassland patches showing a higher abdomen mass. This supports the idea that landscapes with stepping stones, such as grassy margins or woodland/orchards, improve the condition of butterflies. This is also supported by the effect sizes of landscape parameters, with the proportion of woodland/and orchards grassy margin/fallow showing higher values than the proportion of grassland.

## CONCLUSIONS

Our study suggests that landscape features indicative of agricultural intensification favor butterflies with traits enhancing dispersal ability. Overall, the impacts of the surrounding landscape seemed complex, though the configurational heterogeneity appears to be important. In particular, less heterogeneous landscapes selected for more dispersive phenotypes. Interestingly, some trait variation was sex‐dependent, potentially due to their different reproductive strategies. Our results also suggest that adult food stress affects flight propensity (in males), being enhanced if food access is restricted. This phenomenon may also apply to fragmented landscapes with more scattered resources. Based on our findings, we assume that landscapes with low compositional and configurational heterogeneity select for more dispersive phenotypes. Our results thus potentially indicate evolutionary responses to the challenges imposed by intensified agricultural land use. We believe that these findings are important in the context of insect declines in agricultural landscapes and suggest that the impact of landscape effects on intraspecific variation may have been underestimated. Promoting both compositional and configurational heterogeneity through practices such as conserving seminatural habitats and enhancing habitat connectivity may help to support butterfly populations by enabling dispersal between habitat patches and buffering against resource‐related stress.

## AUTHOR CONTRIBUTIONS

**Franziska Deppe**: Writing—original draft; methodology; investigation; formal analysis; data curation; visualization. **Emily Breuer**: Investigation. **Inka Hofmann**: Investigation. **Nicla Koch**: Investigation. **Lara Näckel**: Investigation. **Josua Nowak**: Investigation. **Philip Carlo Plänker**: Investigation. **Anna‐Lena Schmitz**: Investigation. **Lisa Schroeder**: Investigation. **Anna Spitzlei**: Investigation. **Paula Vetter**: Investigation. **Lukas Wassong**: Investigation. **Stefanie Weich**: Investigation. **Michael Weingart**: Investigation. **Luisa Wittkamp**: Investigation. **Mine Yilmazer**: Investigation. **Klaus Fischer**: Writing—review and editing; methodology; supervision; formal analysis; funding acquisition.

## CONFLICT OF INTEREST STATEMENT

The authors declare no conflicts of interest.

## Supporting information


Appendix S1.

## Data Availability

Data (Deppe et al., 2026) are available in Zenodo at https://doi.org/10.5281/zenodo.15229805.
