# Supplementary material for: Landscape heterogeneity affects developmental and dispersal‐related traits of a butterfly in agricultural landscapes
Source: Ecol Appl. 2026 Mar 11;36(2):e70209. doi: 10.1002/eap.70209 (PMC12979024; doi:10.1002/eap.70209)
Supplement: Supplementary file 1 — Appendix S1. [file EAP-36-e70209-s001.pdf]

Appendix S1

Ecological Applications

Landscape heterogeneity affects developmental and dispersal-related traits of a butterfly in agricultural landscapes

Franziska Deppe, Emily Breuer, Inka Hofmann, Nicla Koch, Lara Näckel, Josua Nowak, Philip Carlo Plänker, Anna-Lena Schmitz, Lisa Schroeder, Anna Spitzlei, Paula Vetter, Lukas Wassong, Stefanie Weich, Michael Weingart, Luisa Wittkamp, Mine Yilmazer, Klaus Fischer

Table S1. Proportions of landcover types within 250, 500, and 2000 m radius around the sampling locations. Given is the mean proportion of the land cover types for each of the eight landscapes. Zülpicher Börde, Maifeld, Wetterau and Rheinhessen comprise the 'modern' landscapes and Weyer, Weiler, Grävenwiesbach and Münsterappel comprise the 'traditional' landscapes.

**Landscape**	**Zülpicher Börde**			**Weyer**		
**Spatial scale**	**250 m**	**500 m**	**2000 m**	**250 m**	**500 m**	**2000 m**
Crop fields	62.25	71.27	75.14	18.87	21.35	19.91
Grassland	12.85	8.39	5.92	49.85	42.20	32.14
Fallow	1.70	0.71	1.59	0.32	1.12	0.89
Grassy margins	5.84	4.21	3.40	3.77	3.04	2.68
Forest	9.54	4.74	2.33	15.77	20.21	31.12
Woodland	3.79	3.91	2.18	3.80	3.76	2.90
Orchards	0	0.33	0.21	0.43	0.20	0.21
Water body	0.29	0.23	0.22	0.03	0.01	0.17
Settlements	0.47	3.79	6.86	5.21	6.45	8.23
Vineyard	0	0	0	0	0	0
Roads	3.27	2.40	1.66	1.94	1.67	1.39
Tree nursery	0	0	0.08	0	0	0.01
Fruit plantation	0	0	0.23	0	0	0
Solar	0	0	0	0	0	0.00
Others	0	0	0.18	0	0	0.35
**Landscape**	**Maifeld**			**Weiler**		
**Spatial scale**	**250 m**	**500 m**	**2000 m**	**250 m**	**500 m**	**2000 m**
Crop fields	73.93	79.64	74.35	19.33	19.80	19.92
Grassland	5.60	2.85	3.75	25.17	20.92	21.85
Fallow	5.24	3.70	1.42	0	0.06	0.61
Grassy margins	3.97	3.36	3.46	2.05	2.05	2.00
Forest	1.62	0.95	6.97	45.61	48.97	49.08
Woodland	5.18	4.52	2.85	2.38	1.36	1.64
Orchards	0.01	0.14	0.09	0.33	0.32	0.07
Water body	0	0	0.00	0	0	0.00
Settlements	3.35	3.87	6.20	3.32	5.34	3.82
Vineyard	0	0	0	0	0	0
Roads	1.11	0.95	0.89	1.81	1.18	0.79
Tree nursery	0	0.03	0.01	0	0	0.13
Fruit plantation	0	0	0	0	0	0.03
Solar	0	0	0	0	0	0.06
Others	0	0	0	0	0	0

Landscape	Wetterau			Grävenwiesbach		
Spatial scale	250 m	500 m	2000 m	250 m	500 m	2000 m
Crop fields	64.94	64.52	53.20	42.04	40.64	28.59
Grassland	10.27	9.92	10.60	24.65	20.51	16.66
Fallow	0.12	0.90	0.80	0.00	0.25	0.24
Grassy margins	4.38	4.14	3.50	4.28	3.78	3.79
Forest	4.89	6.14	13.97	17.82	19.73	38.47
Woodland	5.00	3.88	3.40	3.87	3.66	3.29
Orchards	3.72	1.90	1.93	0.01	0.12	0.18
Water body	0.04	0.05	0.17	0.04	0.09	0.24
Settlements	5.11	7.24	11.10	5.93	10.10	7.37
Vineyard	0	0	0	0	0	0
Roads	1.55	1.29	1.18	1.36	1.13	0.98
Tree nursery	0	0.01	0.05	0	0	0.16
Fruit plantation	0	0	0.09	0	0	0.02
Solar	0	0	0	0	0	0.01
Others	0	0	0.01	0	0	0
**Landscape**	**Rheinhessen**			**Münsterappel**		
**Spatial scale**	**250 m**	**500 m**	**2000 m**	**250 m**	**500 m**	**2000 m**
Crop fields	34.73	36.75	40.25	30.91	36.46	42.74
Grassland	9.92	4.88	3.61	30.55	25.24	19.68
Fallow	2.64	1.77	1.52	1.27	0.56	0.39
Grassy margins	4.26	4.39	3.99	4.23	3.91	3.62
Forest	4.55	4.58	3.60	20.84	22.30	23.81
Woodland	6.11	3.99	2.62	8.04	5.93	4.39
Orchards	0.69	0.73	0.23	0.13	0.32	0.17
Water body	0.02	0.04	0.03	0	0	0.01
Settlements	7.58	8.70	10.41	1.97	3.97	3.79
Vineyard	26.89	30.67	30.72	0.14	0.19	0.29
Roads	1.62	1.63	1.06	1.91	1.12	0.79
Tree nursery	0	0.64	0.22	0	0	0
Fruit plantation	0.68	0.60	1.48	0	0	0.33
Solar	0	0	0	0	0	0
Others	0.32	0.65	0.26	0	0	0

Table S2. Coordinates of the 9-12 sampling locations per landscape.

Zülpicher Börde	Weyer	Maifeld	Weiler
50.690977, 6.803129	50.573707, 6.697635	50.283574, 7.366785	50.29978, 7.171048
50.698598, 6.786006	50.574208, 6.65995	50.280437, 7.371311	50.292943, 7.14209
50.69489, 6.775715	50.555236, 6.685883	50.269584, 7.382009	50.291092, 7.126441
50.681605, 6.754666	50.515452, 6.648046	50.272025, 7.363335	50.330173, 7.113256
50.679881, 6.726336	50.52785, 6.62838	50.251063, 7.307275	50.320286, 7.079347
50.725716, 6.760023	50.561123, 6.65575	50.263115, 7.286106	50.287285, 7.107517
50.687806, 6.697489	50.533495, 6.652533	50.272484, 7.291055	50.313657, 7.130148
50.721284, 6.767937	50.557767, 6.62774	50.277356, 7.308976	50.323019, 7.057516
50.703417, 6.742665	50.55221, 6.606003	50.292971, 7.388719	50.327194, 7.055235
50.716368, 6.733778	50.521512, 6.588713		50.332948, 7.082621
50.728201, 6.829692			
50.734537, 6.760592			
**Wetterau**	**Grävenwiesbach**	**Rheinhessen**	**Münsterappel**
50.410593, 8.732806	50.361052, 8.461266	49.775908, 7.987749	49.715255, 7.878309
50.416588, 8.728312	50.373856, 8.375296	49.8195811, 7.9216224	49.7314565, 7.8745037
50.421289, 8.751811	50.373329, 8.409172	49.812566, 8.0144707	49.734394, 7.845756
50.372347, 8.626665	50.373936, 8.420418	49.8113805, 7.9392636	49.742354, 7.889782
50.387579, 8.632516	50.382859, 8.405653	49.7996646, 7.942328	49.727197, 7.885675
50.378892, 8.647614	50.38729, 8.475178	49.7870081, 7.9833152	49.7429134, 7.9026008
50.387258, 8.756054	50.373909, 8.458822	49.7781337, 8.0185188	49.756246, 7.895375
50.36898, 8.646368	50.365426, 8.437199	49.7894681, 8.0345095	49.7518256, 7.8824356
50.400932, 8.75752	50.353001, 8.434226	49.7915408, 7.9365814	49.7223971, 7.8459525
50.395363, 8.630057	50.3804, 8.461653		49.740069, 7.8824282
	50.357176, 8.394349		49.714503, 7.890198

Table S3. Data transformation methods applied to trait variables by the bestNormalize function.

Trait variable	Date transformation
**Offspring**	
Egg development time - female	Standardized double reversed Log_b(x + a) Transformation
Larval time - female	Standardized asinh(x) Transformation
Pupal time - female	Standardized sqrt(x + a) Transformation
Pupal mass - female	center_scale(x) Transformation
Larval growth rate - female	Standardized Box Cox Transformation
Thorax mass - female	orderNorm Transformation
Thorax-abdomen ratio - female	orderNorm Transformation
Forewing length - female	Standardized sqrt(x + a) Transformation
Forewing area - female	Standardized Box Cox Transformation
Wing loading - female	orderNorm Transformation
Wing aspect ratio - female	Standardized sqrt(x + a) Transformation
Flight propensity - female	Standardized sqrt(x + a) Transformation
Flight capacity - female	orderNorm Transformation
Abdomen mass - female	Standardized Box Cox Transformation
Relative fat content - female	Standardized asinh(x) Transformation
Egg development time - male	Standardized double reversed Log_b(x + a)
Larval time - male	orderNorm Transformation
Pupal time - male	center_scale(x) Transformation
Pupal mass - male	Standardized sqrt(x + a) Transformation
Larval growth rate - male	Standardized asinh(x) Transformation
Thorax mass - male	orderNorm Transformation
Thorax-abdomen ratio - male	Standardized Yeo-Johnson Transformation
Forewing length - male	Standardized sqrt(x + a) Transformation
Forewing area - male	Standardized Box Cox Transformation
Wing loading - male	orderNorm Transformation
Wing aspect ratio - male	Standardized double reversed Log_b(x + a) Transformation
Flight propensity - male	orderNorm Transformation
Flight capacity - male	orderNorm Transformation
Abdomen mass - male	Standardized Box Cox Transformation
Relative fat content - male	orderNorm Transformation
**Field-caught females**	
Thorax mass	center_scale(x) Transformation
Thorax abdomen ratio	center_scale(x) Transformation
Forewing length	center_scale(x) Transformation
Forewing area	center_scale(x) Transformation
Wing loading	Standardized sqrt(x + a)
Wing aspect ratio	Standardized asinh(x) Transformation

Abdomen mass	Standardized Yeo-Johnson Transformation
Relative fat content	orderNorm Transformation

Table S4. List of the landscape parameters assessed at three spatial scales (250, 500, 2000 m). Parameters marked with 'x' were excluded for model averaging in the respective spatial scale for both field-caught females and their offspring due to strong correlations with r > |0.7|.

**Landscape parameter**	**250 m**	**500 m**	**2000 m**
Habitat diversity			
Crop fields (%)		x	x
Grassland (%)			
Grassy margin / Fallow (%)			
Forest (%)			
Woodland / Orchards (%)			
PAR crop fields			
PAR grassland			
Mean nearest distance among grassland patches	x		x
Nearest habitat patch distance			
Patch size of sampling location			

Table S5. Means and SE for all traits investigated in *C. pamphilus* offspring, separated by sex.

	Female		Male	
**Trait**	**Mean**	**SE**	**Mean**	**SE**
Egg development time (days)	8.82	0.06	8.70	0.06
Larval time (days)	36.89	0.32	32.34	0.27
Pupal time (days)	9.86	0.07	10.31	0.07
Pupal mass (mg)	86.65	12.25	67.75	10.24
Larval growth rate (mg/day)	0.13	0.002	0.13	0.001
Thorax mass (mg)	10.92	0.11	7.86	0.08
Thorax-abdomen ratio	0.62	0.01	1.88	0.02
Forewing length (mm)	15.28	0.04	14.39	0.04
Forewing area (mm^2)	98.22	0.50	87.58	0.39
Wing loading (mg/mm)	0.36	0.003	0.20	0.002
Wing aspect ratio	9.55	0.03	9.49	0.03
Flight propensity (min)	78.12	1.39	72.63	1.57
Flight capacity (sec)	30.01	0.91	36.53	1.10
Abdomen mass (mg)	18.74	0.28	4.50	0.09
Relative fat content (%)	5.22	0.14	11.12	0.34

Table S6. Means and SE for all traits investigated in *C. pamphilus* offspring, separated by landscape type (modern versus traditional).

	Modern		Traditional	
**Trait**	**Mean**	**SE**	**Mean**	**SE**
Egg development time (days)	8.71	0.06	8.81	0.06
Larval time (days)	34.71	0.30	34.22	0.32
Pupal time (days)	10.24	0.06	9.94	0.07
Pupal mass (mg)	78.03	0.62	75.04	0.72
Larval growth rate (mg/day)	0.13	0.001	0.13	0.001
Thorax mass (mg)	9.56	0.11	8.96	0.13
Thorax-abdomen ratio	1.32	0.03	1.27	0.04
Forewing length (mm)	14.91	0.04	14.68	0.05
Forewing area (mm^2)	93.32	0.41	91.61	0.56
Wing loading (mg/mm)	0.28	0.004	0.27	0.005
Wing aspect ratio	9.56	0.03	9.46	0.03
Flight propensity (min)	74.77	1.44	75.69	1.59
Flight capacity (sec)	34.71	0.97	31.99	1.12
Abdomen mass (mg)	11.25	0.37	10.98	0.4
Relative fat content (%)	8.22	0.27	8.55	0.35

Table S7. Effects of landscape parameters (fixed; measured in three different radii around the sampling location), year and generation (nested in year) on various traits in female and male offspring of *Coenonympha pamphilus* with the random factors replicate landscape pairs, location (nested in replicate landscape pairs) and family, i.e. all offspring of one individual female (nested in location and replicate landscape pairs). For traits with multiple models within ΔAICc ≤ 2, model-averaged results are shown (z values) and for traits where only one model was supported, estimates are from the single best mixed model (t values with df). Models are shown if at least one landscape parameter was significant during the model averaging process. Significant p-values are given in bold. PAR: Perimeter to area ratio.

Trait/factor	Estimate	SE	Test	z-value / t-value	df	p
**Egg development time - male (250 m)**						
Grassland (%)	0.159	0.07	z	2.182		**0.0291**
Year	0.307	0.15		2.029		**0.0424**
Generation (within year 1)	0.279	0.22		1.285		0.1989
Generation (within year 2)	-0.903	0.32		2.847		**0.0044**
**Egg development time - male (500 m)**						
Grassland (%)	0.183	0.07	z	2.463		**0.0138**
**Larval time - female (250 m)**						
Habitat diversity	0.126	0.05	z	2.298		**0.0215**
Woodland / Orchards (%)	0.154	0.06		2.638		**0.0083**
Year	1.307	0.13		10.092		**< 0.0001**
Generation (within year 1)	0.473	0.20		2.374		**0.0176**
Generation (within year 2)	0.889	0.25		3.51		**0.0004**
**Larval time - female (500 m)**						
Habitat diversity	0.137	0.06	z	2.462		**0.0138**
Woodland / Orchards (%)	0.144	0.06		2.602		**0.0093**
Year	1.289	0.12		10.387		**< 0.0001**
Generation (within year 1)	0.427	0.19		2.228		**0.0259**
Generation (within year 2)	0.892	0.26		3.382		**0.0007**
**Larval time - female (2000 m)**						
Grassland (%)	-0.152	0.06	z	2.562		**0.0104**
Habitat diversity	0.202	0.07		3.013		**0.0026**
Year	1.317	0.12		11.067		**< 0.0001**
Generation (within year 1)	0.384	0.18		2.174		**0.0297**
Generation (within year 2)	0.926	0.25		3.764		**0.0002**
**Larval time - male (2000 m)**						
Forest (%)	-0.112	0.06	z	2.009		**0.0445**
Year	1.029	0.13		7.86		**< 0.0001**
Generation (within year 1)	0.420	0.18		2.388		**0.0169**
Generation (within year 2)	1.070	0.24		4.5		**0.0000**
**Pupal time - female (2000 m)**						
Grassland (%)	-0.146	0.07	z	2.194		**0.0282**
Habitat diversity	-0.132	0.07		1.974		**0.0483**
**Pupal mass - female (500 m)**						
Grassland (%)	-0.199	0.10	z	2.08		**0.0375**
Year	-0.074	0.14		0.532		0.5951
Generation (within year 1)	-0.746	0.22		3.33		**0.0009**
Generation (within year 2)	-0.222	0.32		0.699		0.4843
**Pupal mass - female (2000 m)**						
PAR grassland	0.229	0.06	t	3.825	12.74	**0.0022**
Year	-0.021	0.13		-0.157	73.01	0.8753
Generation (within year 1)	-0.722	0.20		-3.548	74.53	**0.0007**
Generation (within year 2)	-0.202	0.29		-0.693	276.27	0.4887

**Pupal mass - male (250 m)**						
Grassland (%)	-0.132	0.07		2.014		**0.0440**
Year	-0.016	0.14	z	0.119		0.9051
Generation (within year 1)	-1.052	0.20		5.365		**< 0.0001**
Generation (within year 2)	-1.166	0.29		4.074		**< 0.0001**
**Pupal mass - male (500 m)**						
Grassland (%)	-0.129	0.07		1.91		0.0562
Year	-0.019	0.14	z	0.142		0.8869
Generation (within year 1)	-1.050	0.20		5.354		**< 0.0001**
Generation (within year 2)	-0.938	0.26		3.576		**0.0003**
**Larval growth rate - female (250 m)**						
Woodland / Orchards (%)	-0.136	0.06		2.348		**0.0189**
Year	-1.287	0.13	z	9.983		**< 0.0001**
Generation (within year 1)	-0.696	0.20		3.56		**0.0004**
Generation (within year 2)	-0.887	0.25		3.478		**0.0005**
**Larval growth rate - female (500 m)**						
Habitat diversity	-0.132	0.06		2.361		**0.0182**
Woodland / Orchards (%)	-0.134	0.06		2.425		**0.0153**
Year	-1.279	0.12	z	10.347		**< 0.0001**
Generation (within year 1)	-0.641	0.19		3.279		**0.0010**
Generation (within year 2)	-0.866	0.26		3.26		**0.0011**
**Larval growth rate - female (2000 m)**						
Grassland (%)	0.118	0.06		1.985		**0.0472**
Habitat diversity	-0.171	0.06		2.807		**0.0050**
Year	-1.301	0.12	z	10.677		**< 0.0001**
Generation (within year 1)	-0.596	0.18		3.252		**0.0011**
Generation (within year 2)	-0.878	0.25		3.515		**0.0004**
**Thorax mass - female (250 m)**						
Grassland (%)	-0.155	0.07		2.126		**0.0335**
Year	-0.293	0.14	z	2.156		**0.0311**
Generation (within year 1)	-0.626	0.21		3		**0.0027**
Generation (within year 2)	-0.545	0.30		1.784		0.0745
**Thorax mass - female (500 m)**						
Grassland (%)	-0.166	0.08		2.097		**0.0360**
Year	-0.319	0.13	z	2.457		**0.0140**
Generation (within year 1)	-0.654	0.21		3.178		**0.0015**
Generation (within year 2)	-0.693	0.31		2.208		**0.0273**
**Thorax mass - female (2000 m)**						
PAR grassland	0.248	0.05		4.648	99.35	**< 0.0001**
Year	-0.249	0.12	t	-2.095	86.28	**0.0391**
Generation (within year 1)	-0.568	0.18		-3.126	111.17	**0.0023**
Generation (within year 2)	-0.644	0.28		-2.264	370.96	**0.0242**
**Thorax mass - male (500 m)**						
Grassy margin / Fallow (%)	0.081	0.05		1.585		0.1130
Habitat diversity	-0.162	0.06		2.509		**0.0121**
Mean nearest distance among grassland patches	0.122	0.07		1.661		0.0967
Nearest habitat patch distance	-0.051	0.07		0.754		0.4509
PAR crop fields	-0.136	0.05		2.826		**0.0047**
PAR grassland	-0.125	0.05	z	2.355		**0.0185**
Sampling location patch size	-0.038	0.07		0.512		0.6086
Woodland / Orchards (%)	0.205	0.07		3.077		**0.0021**
Year	-0.287	0.10		2.967		**0.0030**
Generation (within year 1)	-0.578	0.16		3.504		**0.0005**
Generation (within year 2)	-0.017	0.25		0.066		0.9470

**Thorax-abdomen ratio - female (250 m)**						
Crop fields (%)	0.024	0.11		0.219		0.8264
Forest (%)	-0.105	0.05		2.192		**0.0284**
Grassland (%)	-0.094	0.06		1.659		0.0970
Grassy margin / Fallow (%)	0.056	0.05		1.169		0.2423
Nearest habitat patch distance	0.079	0.05		1.645		0.1000
PAR crop fields	0.058	0.05	z	1.221		0.2223
PAR grassland	0.038	0.04		0.862		0.3886
Sampling location patch size	0.076	0.05		1.675		0.0940
Woodland / Orchards (%)	0.033	0.05		0.679		0.4972
Year	0.846	0.10		8.677		**< 0.0001**
Generation (within year 1)	0.432	0.16		2.752		**0.0059**
Generation (within year 2)	0.472	0.27		1.758		0.0787
**Thorax-abdomen ratio - female (500 m)**						
Forest (%)	-0.089	0.04		2.015		**0.0439**
Grassland (%)	-0.108	0.06		1.834		0.0666
Grassy margin / Fallow (%)	-0.018	0.05		0.376		0.7069
Habitat diversity	0.046	0.06		0.824		0.4100
Mean nearest distance among grassland patches	0.056	0.04		1.341		0.1801
Nearest habitat patch distance	0.065	0.05		1.44		0.1499
PAR crop fields	-0.071	0.05	z	1.525		0.1272
PAR grassland	-0.026	0.05		0.584		0.5594
Sampling location patch size	0.076	0.04		1.813		0.0698
Woodland / Orchards (%)	0.019	0.04		0.43		0.6674
Year	0.883	0.09		9.454		**< 0.0001**
Generation (within year 1)	0.477	0.16		3.03		**0.0025**
Generation (within year 2)	0.416	0.28		1.486		0.1373
**Thorax-abdomen ratio - female (2000 m)**						
Forest (%)	-0.047	0.05		0.994		0.3202
Grassland (%)	0.019	0.05		0.369		0.7122
Habitat diversity	0.041	0.06		0.715		0.4747
Nearest habitat patch distance	0.094	0.05		1.732		0.0832
PAR grassland	0.127	0.05		2.368		**0.0179**
Sampling location patch size	0.057	0.04	z	1.387		0.1656
Woodland / Orchards (%)	-0.120	0.06		2.162		**0.0306**
Year	0.849	0.10		8.738		**< 0.0001**
Generation (within year 1)	0.602	0.15		4.027		**0.0001**
Generation (within year 2)	0.447	0.27		1.674		0.0941
**Forewing length - female (250 m)**						
Crop fields (%)	0.172	0.07		2.589		**0.0096**
Forest (%)	-0.138	0.05		2.511		**0.0120**
Year	0.316	0.13	z	2.472		**0.0134**
Generation (within year 1)	-0.851	0.20		4.264		**< 0.0001**
Generation (within year 2)	-1.048	0.30		3.451		**0.0006**
**Forewing length - female (500 m)**						
Grassland (%)	-0.140	0.06		2.184		**0.0290**
Year	0.308	0.13		2.453		**0.0142**
Generation (within year 1)	-0.986	0.19	z	5.128		**< 0.0001**
Generation (within year 2)	-1.199	0.31		3.807		**0.0001**
**Forewing length - female (2000 m)**						
Grassland (%)	-0.157	0.06		2.812		**0.0049**
PAR grassland	0.146	0.05		2.658		**0.0079**
Year	0.326	0.13	z	2.56		**0.0105**
Generation (within year 1)	-0.946	0.19		5.064		**< 0.0001**
Generation (within year 2)	-1.095	0.30		3.668		**0.0002**
**Forewing area - female (250 m)**						
Crop fields (%)	0.147	0.07		2.017		**0.0437**
Forest (%)	-0.148	0.06		2.493		**0.0127**
Year	-0.124	0.14	z	0.888		0.3743
Generation (within year 1)	-0.915	0.21		4.366		**< 0.0001**
Generation (within year 2)	-1.037	0.32		3.281		**0.0010**

**Forewing area - female (2000 m)**						
PAR grassland	0.169	0.06	t	2.863	92.79	**0.0052**
Year	-0.047	0.13		-0.354	80.26	0.7243
Generation (within year 1)	-0.904	0.20		-4.569	98.50	**< 0.0001**
Generation (within year 2)	-1.165	0.31		-3.763	331.18	0.0002
**Wing loading - female (500 m)**						
Forest (%)	0.030	0.04	z	0.691		0.4898
Grassland (%)	-0.067	0.06		1.2		0.2300
Grassy margin / Fallow (%)	-0.084	0.05		1.79		0.0735
Habitat diversity	0.048	0.07		0.696		0.4866
Mean nearest distance among grassland patches	-0.056	0.05		1.113		0.2655
Nearest habitat patch distance	0.062	0.06		1.086		0.2775
PAR grassland	0.064	0.05		1.347		0.1779
Sampling location patch size	-0.060	0.04		1.403		0.1605
Woodland / Orchards (%)	0.108	0.05		2.216		**0.0267**
Year	-0.969	0.09		10.455		**< 0.0001**
Generation (within year 1)	-0.424	0.15		2.727		**0.0064**
Generation (within year 2)	-0.248	0.30		0.822		0.4108
**Wing loading - female (2000 m)**						
Grassland (%)	-0.092	0.05	z	1.887		0.0591
Grassy margin / Fallow (%)	-0.042	0.05		0.832		0.4054
Habitat diversity	-0.019	0.06		0.328		0.7428
Nearest habitat patch distance	0.032	0.05		0.655		0.5124
PAR crop fields	-0.039	0.05		0.86		0.3898
PAR grassland	0.071	0.06		1.225		0.2207
Sampling location patch size	-0.019	0.04		0.474		0.6353
Woodland / Orchards (%)	0.116	0.05		2.241		**0.0250**
Year	-0.935	0.10		9.81		**< 0.0001**
Generation (within year 1)	-0.454	0.15		3.08		**0.0021**
Generation (within year 2)	-0.273	0.29		0.952		0.3412
**Wing loading - male (250 m)**						
Grassland (%)	-0.118	0.06	z	2.01		**0.0445**
Year	-0.597	0.12		5.168		**< 0.0001**
Generation (within year 1)	-0.361	0.18		2.044		**0.0409**
Generation (within year 2)	0.144	0.30		0.483		0.6290
**Wing loading - male (2000 m)**						
Forest (%)	-0.104	0.05	z	2.131		**0.0331**
Grassland (%)	-0.139	0.05		2.638		**0.0083**
Habitat diversity	-0.114	0.05		2.211		**0.0270**
PAR grassland	0.145	0.05		2.638		**0.0083**
Year	-0.622	0.13		4.891		**< 0.0001**
Generation (within year 1)	-0.439	0.16		2.688		**0.0072**
Generation (within year 2)	0.211	0.26		0.821		0.4118
**Flight propensity - female (250 m)**						
Crop fields (%)	0.075	0.05	z	1.608		0.1079
Forest (%)	-0.038	0.04		1.022		0.3067
Grassland (%)	-0.043	0.05		0.807		0.4198
Grassy margin / Fallow (%)	0.022	0.04		0.526		0.5986
Habitat diversity	-0.046	0.05		0.978		0.3282
Nearest habitat patch distance	0.139	0.05		3.006		**0.0027**
PAR crop fields	0.032	0.04		0.878		0.3801
Sampling location patch size	0.023	0.04		0.557		0.5778
Woodland / Orchards (%)	0.034	0.05		0.709		0.4784
Year	0.172	0.09		1.825		0.0680
Generation (within year 1)	0.205	0.15		1.35		0.1771
**Flight propensity - female (500 m)**						
Forest (%)	-0.018	0.06	z	0.285		0.7760
Grassland (%)	-0.095	0.08		1.182		0.2374
Habitat diversity	-0.142	0.07		1.961		**0.0499**
Mean nearest distance among grassland patches	0.057	0.06		0.871		0.3836
Nearest habitat patch distance	0.144	0.07		2.022		**0.0432**
PAR grassland	0.050	0.07		0.746		0.4557
Sampling location patch size	0.056	0.06		0.978		0.3280
Woodland / Orchards (%)	0.085	0.08		1.119		0.2633
Year	0.286	0.13		2.132		**0.0330**
Generation (within year 1)	0.310	0.21		1.457		0.1451

**Flight propensity - female (2000 m)**						
Forest (%)	-0.066	0.06	z	1.069		0.2849
Grassland (%)	-0.077	0.06		1.189		0.2344
Habitat diversity	-0.099	0.07		1.432		0.1520
Nearest habitat patch distance	0.167	0.07		2.447		**0.0144**
PAR grassland	0.025	0.07		0.37		0.7117
Sampling location patch size	0.054	0.06		0.928		0.3533
Year	0.260	0.12		2.133		**0.0329**
Generation (within year 1)	0.182	0.19		0.949		0.3424
**Flight capacity - female (2000 m)**						
Nearest habitat patch distance	-0.162	0.07	z	2.357		**0.0184**
Habitat diversity	-0.116	0.07		1.564		0.1177
Woodland / Orchards (%)	-0.120	0.07		1.708		0.0877
Grassland (%)	-0.064	0.07		0.975		0.3297
Grassy margin / Fallow (%)	0.063	0.05		1.14		0.2543
Forest (%)	-0.054	0.06		0.876		0.3809
PAR grassland	-0.049	0.07		0.723		0.4699
Sampling location patch size	-0.032	0.05		0.576		0.5646
Year	0.237	0.13		1.828		0.0676
Generation (within year 1)	-0.521	0.20		2.636		**0.0084**
**Flight capacity - male (500 m)**						
Forest (%)	-0.027	0.06	z	0.479		0.6321
Grassland (%)	-0.106	0.06		1.668		0.0953
Mean nearest distance among grassland patches	0.082	0.07		1.211		0.2258
PAR crop fields	-0.140	0.07		2.006		**0.0449**
PAR grassland	-0.146	0.05		2.695		**0.0070**
Sampling location patch size	-0.088	0.06		1.355		0.1753
Woodland / Orchards (%)	0.021	0.05		0.397		0.6917
Year	-0.113	0.11		1.023		0.3065
Generation (within year 1)	-0.508	0.17		2.903		**0.0037**
**Abdomen mass - female (500 m)**						
Mean nearest distance among grassland patches	-0.098	0.05	z	2.083		**0.0372**
Nearest habitat patch distance	-0.110	0.05		2.144		**0.0320**
Year	-0.976	0.11		8.813		**< 0.0001**
Generation (within year 1)	-0.828	0.17		4.783		**< 0.0001**
Generation (within year 2)	-0.827	0.27		3.032		**0.0024**
**Abdomen mass - female (2000 m)**						
PAR grassland	0.147	0.05	t	2.996	84.74	**0.0036**
Year	-0.933	0.11		-8.565	72.71	**< 0.0001**
Generation (within year 1)	-0.806	0.17		-4.847	94.19	**< 0.0001**
Generation (within year 2)	-0.853	0.26		-3.286	351.94	0.0011
**Abdomen mass - male (500 m)**						
Grassy margin / Fallow (%)	0.110	0.05	z	2.309		**0.0210**
Year	-0.821	0.12		7.049		**< 0.0001**
Generation (within year 1)	-0.485	0.17		2.824		**0.0047**
Generation (within year 2)	-0.074	0.25		0.299		0.7652
**Abdomen mass - male (2000 m)**						
Grassland (%)	-0.107	0.05	z	2.127		**0.0334**
Year	-0.863	0.11		7.675		**< 0.0001**
Generation (within year 1)	-0.583	0.16		3.654		0.0003
Generation (within year 2)	-0.038	0.24		0.157		0.8749

Table S8. Means and SE for all traits investigated in field-caught females of *C. pamphilus* offspring, separated by landscape type (modern and traditional).

	Modern		Traditional	
**Trait**	**Mean**	**SE**	**Mean**	**SE**
Thorax mass (mg)	8.68	0.46	8.59	0.41
Thorax-abdomen ratio	0.95	0.04	0.93	0.04
Forewing length (mm)	15.75	0.08	15.67	0.11
Forewing area (mm^2)	104.56	1.05	101.92	1.36
Wing loading (mg/mm)	0.23	0.01	0.24	0.01
Wing aspect ratio	9.53	0.06	9.67	0.05
Abdomen mass (mg)	10.32	0.73	10.49	0.64
Relative fat content (%)	6.24	0.52	6.33	0.55

Table S9. Effects of landscape parameters (fixed; measured in three different radii around the sampling location), year and generation (nested in year) on various traits in field-caught females of *Coenonympha pamphilus* with the random factors replicate landscape pairs and location (nested in replicate landscape pairs). For traits with multiple models within ΔAICc ≤ 2, model-averaged results are shown (z values) and for traits where only one model was supported, estimates are from the single best mixed model (t values with df). Models are shown if at least one landscape parameter was significant during the model averaging process. Significant p-values are given in bold. PAR: Perimeter to area ratio.

**Trait/factor**	**Estimate**	**SE**	**z-value**	**p**
**Thorax mass (500 m)**				
Grassland (%)	0.088	0.09	1.010	0.3123
Habitat diversity	0.081	0.08	1.031	0.3023
Mean nearest distance among grassland patches	-0.065	0.08	0.861	0.3893
PAR crop fields	0.235	0.08	2.973	**0.0030**
PAR grassland	-0.087	0.08	1.143	0.2531
Year	-1.072	0.18	5.976	< **0.0001**
Generation (within year 1)	-0.881	0.27	3.189	0.0014
Generation (within year 2)	-0.070	0.23	0.308	0.7581
**Thorax-abdomen ratio (250 m)**				
Grassy margin / Fallow (%)	0.179	0.08	2.161	**0.0307**
Nearest habitat patch distance	0.056	0.08	0.671	0.5020
PAR crop fields	-0.066	0.08	0.784	0.4333
PAR grassland	0.094	0.08	1.167	0.2432
Sampling location patch size	0.067	0.08	0.810	0.4177
Woodland / Orchards (%)	0.047	0.08	0.563	0.5735
Year	0.584	0.17	3.367	**0.0008**
Generation (within year 1)	0.437	0.29	1.484	0.1377
Generation (within year 2)	0.173	0.24	0.699	0.4843
**Thorax-abdomen ratio (2000 m)**				
Grassland (%)	0.044	0.09	0.487	0.6264
Grassy margin / Fallow (%)	0.170	0.08	2.062	**0.0392**
Habitat diversity	0.036	0.08	0.442	0.6583
PAR crop fields	-0.093	0.08	1.144	0.2526
PAR grassland	-0.050	0.08	0.629	0.5292
Sampling location patch size	0.079	0.08	0.936	0.3491
Woodland / Orchards (%)	-0.046	0.08	0.556	0.5785
Year	0.626	0.17	3.586	**0.0003**
Generation (within year 1)	0.415	0.27	1.538	0.1240
Generation (within year 2)	0.263	0.23	1.137	0.2556

**Forewing area (2000 m)**				
Grassland (%)	-0.171	0.09	1.961	**0.0499**
Grassy margin / Fallow (%)	0.179	0.09	1.987	**0.0469**
PAR grassland	0.163	0.09	1.830	0.0672
Year	-0.593	0.18	3.183	**0.0015**
Generation (within year 1)	-1.025	0.29	3.540	**0.0004**
Generation (within year 2)	-0.082	0.23	0.356	0.7216
**Wing loading (500 m)**				
Grassland (%)	0.187	0.09	2.118	**0.0341**
PAR crop fields	0.188	0.08	2.369	**0.0178**
PAR grassland	-0.118	0.08	1.500	0.1335
Habitat diversity	0.049	0.09	0.556	0.5785
Mean nearest distance among grassland patches	-0.073	0.07	0.970	0.3320
Nearest habitat patch distance	0.105	0.09	1.100	0.2713
Woodland / Orchards (%)	0.099	0.08	1.188	0.2348
Year	-1.162	0.18	6.529	< **0.0001**
Generation (within year 1)	-0.833	0.27	3.102	**0.0019**
Generation (within year 2)	-0.073	0.22	0.325	0.7452
**Wing aspect ratio (2000 m)**				
Habitat diversity	0.101	0.08	1.200	0.2302
Nearest habitat patch distance	-0.049	0.09	0.561	0.5751
PAR crop fields	0.147	0.09	1.638	0.1014
PAR grassland	-0.270	0.09	2.922	**0.0035**
Woodland / Orchards (%)	0.065	0.09	0.714	0.4753
Year	0.666	0.17	3.846	**0.0001**
Generation (within year 1)	0.480	0.27	1.773	0.0763
Generation (within year 2)	0.066	0.22	0.295	0.7678
**Abdomen mass (500 m)**				
Grassland (%)	0.180	0.08	2.129	**0.0333**
Mean nearest distance among grassland patches	-0.123	0.09	1.380	0.1676
Nearest habitat patch distance	0.109	0.09	1.144	0.2528
PAR crop fields	0.152	0.08	1.999	**0.0456**
PAR grassland	-0.118	0.07	1.560	0.1188
Woodland / Orchards (%)	0.046	0.07	0.623	0.5333
Year	-1.253	0.17	7.332	< **0.0001**
Generation (within year 1)	-0.917	0.26	3.534	**0.0004**
Generation (within year 2)	-0.159	0.22	0.729	0.4661
**Relative fat content (250 m)**				
Nearest habitat patch distance	0.135	0.09	1.566	0.1173
PAR grassland	0.109	0.08	1.418	0.1561
Woodland / Orchards (%)	0.226	0.09	2.571	**0.0101**
Year	-0.609	0.18	3.320	**0.0009**
Generation (within year 1)	-0.709	0.27	2.565	**0.0103**
Generation (within year 2)	-0.470	0.23	2.038	**0.0416**
**Relative fat content (500 m)**				
Grassy margin / Fallow (%)	-0.225	0.08	2.75	**0.0060**
Nearest habitat patch distance	0.172	0.09	1.94	0.0526
PAR crop fields	-0.072	0.08	0.92	0.3598
Sampling location patch size	-0.062	0.08	0.73	0.4660

Woodland / Orchards (%)	0.329	0.09	3.48	**0.0005**
Year	-0.613	0.18	3.43	**0.0006**
Generation (within year 1)	-0.792	0.27	2.94	**0.0033**
Generation (within year 2)	-0.431	0.22	1.93	0.0538
**Relative fat content (2000 m)**				
Forest (%)	0.090	0.08	1.12	0.2616
Grassy margin / Fallow (%)	-0.070	0.08	0.91	0.3640
Nearest habitat patch distance	0.112	0.08	1.32	0.1867
Woodland / Orchards (%)	0.207	0.08	2.47	**0.0137**
Year	-0.577	0.18	3.12	**0.0018**
Generation (within year 1)	-0.636	0.26	2.47	**0.0135**
Generation (within year 2)	-0.461	0.22	2.10	**0.0359**

Table S10. Overview of landscape parameters significantly affecting butterfly traits of *C. pamphilus*, separated by field-caught females and female and male offspring. Overlaps of landscape parameters within one trait between the two generations are marked orange. PAR: Perimeter to area ratio.

**Trait**	**Field-caught**	**Offspring**	
	**Female (n = 144)**	**Male (n = 511)**	**Female (n = 462)**
Thorax mass	PAR crop fields	PAR crop fields	Grassland (%)
		Habitat diversity	PAR grassland
		PAR grassland	
		Woodland / Orchards (%)	
Thorax-abdomen ratio	Grassy margin / Fallow (%)		Forest (%)
			PAR grassland
			Woodland / Orchards (%)
Forewing length			Crop fields (%)
			Forest (%)
			Grassland (%)
			PAR grassland
Forewing area	Grassland (%)		Crop fields (%)
	Grassy margin / Fallow (%)		Forest (%)
			PAR grassland
Wing loading	PAR crop fields	Forest (%)	Woodland / Orchards (%)
	Grassland (%)	Grassland (%)	
		Habitat diversity	
		PAR grassland	
Wing aspect ratio	PAR grassland		
Flight propensity	not measured		Habitat diversity
			Nearest habitat patch distance
Flight capacity	not measured	PAR crop fields	Nearest habitat patch distance
		PAR grassland	
Abdomen mass	PAR crop fields	Grassland (%)	Mean nearest distance among grassland patches
		Grassy margin / Fallow (%)	Nearest habitat patch distance
			PAR grassland
Relative fat content	Grassy margin / Fallow (%)		
	Woodland / Orchards (%)		

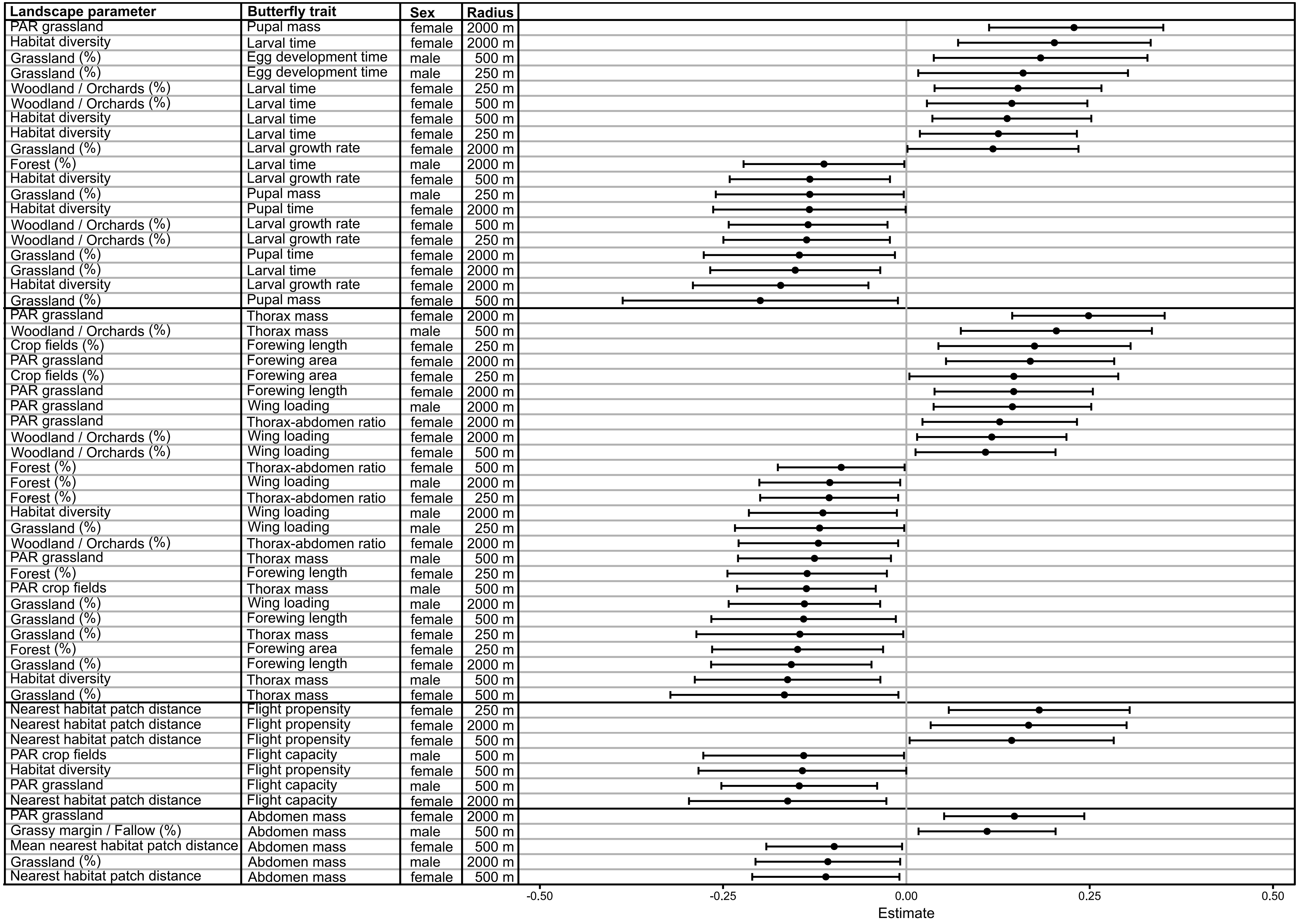

Figure S1. Model-averaged parameter estimates and 95 % confidence intervals of the effect of standardized landscape parameters on traits of offspring of *C. pamphilus*. Separate models were fitted for each sex and spatial scale.
